# Upregulation of breathing rate during running exercise by central locomotor circuits in mice

**DOI:** 10.1038/s41467-023-38583-6

**Published:** 2023-05-22

**Authors:** Coralie Hérent, Séverine Diem, Giovanni Usseglio, Gilles Fortin, Julien Bouvier

**Affiliations:** 1 Université Paris-Saclay, CNRS, Institut des Neurosciences Paris-Saclay, 91400 Saclay, France; 2 Institut de Biologie de l’École Normale Supérieure (IBENS), École Normale Supérieure, CNRS, INSERM, PSL Research University, 75005 Paris, France; 3 Present Address: Champalimaud Research, Champalimaud Foundation, 1400-038 Lisbon, Portugal; 4 Present Address: Institute of Functional Genomics, University of Montpellier, CNRS, INSERM, 34094 Montpellier, France

**Keywords:** Central pattern generators, Spinal cord, Neural circuits, Respiration

## Abstract

While respiratory adaptation to exercise is compulsory to cope with the increased metabolic demand, the neural signals at stake remain poorly identified. Using neural circuit tracing and activity interference strategies in mice, we uncover here two systems by which the central locomotor network can enable respiratory augmentation in relation to running activity. One originates in the mesencephalic locomotor region (MLR), a conserved locomotor controller. Through direct projections onto the neurons of the preBötzinger complex that generate the inspiratory rhythm, the MLR can trigger a moderate increase of respiratory frequency, prior to, or even in the absence of, locomotion. The other is the lumbar enlargement of the spinal cord containing the hindlimb motor circuits. When activated, and through projections onto the retrotrapezoid nucleus (RTN), it also potently upregulates breathing rate. On top of identifying critical underpinnings for respiratory hyperpnea, these data also expand the functional implication of cell types and pathways that are typically regarded as “locomotor” or “respiratory” related.

## Abstract (web summary)

The neuronal basis for respiratory augmentation during running is poorly understood. Here, the authors identify two neuronal pathways by which the central locomotor network can upregulate respiratory rate in running mice.

## Introduction

Breathing is a vital behavior that must combine extreme robustness with continuous adaptability. One striking example is the abrupt augmentation of ventilation at the transition from rest to running in order to maintain homeostasis in spite of increased metabolic demand^1^. This “exercise hyperpnoea” is manifested by an increase in both respiratory frequency and volume. It has long been proposed that its main trigger, at least for acute exercise, is of neuronal nature, i.e., relies on activatory signals from locomotor effectors or circuits impacting onto the respiratory generator in the brainstem^1–3^. However, the underlying cells and circuits are not fully elucidated.

Running hyperpnea can occur without temporal synchronization of breathes to strides^4,5^, in the absence of peripheral signals^6^, or during mental simulation of exercise^7–9^, highlighting that its main neuronal trigger may be of central, rather than peripheral, origin. Of particular interest are therefore brain regions that command or execute locomotor movements and could provide a parallel drive to respiratory centers^10,11^. The mesencephalic locomotor region (MLR) in the dorsal midbrain is considered the main site of locomotor initiation throughout the animal kingdom, likely including humans^12–14^. Stimulation of the MLR, and particularly its cuneiform nucleus (CnF) component, engages forward locomotion at a speed that is commensurate to the intensity of the stimulus^15–19^, making it a candidate neuronal encoder and driver of running intensity. Interestingly, the MLR may also upregulate breathing activity^10^ and work in the lamprey, an ancestral vertebrate, recently argued for a parallel connectivity of this structure to respiratory centers^20^. This however remains to be investigated in terrestrial mammals. Another central drive to respiratory centers may originate in the circuits of the spinal cord that elaborate the locomotor rhythm and coordinate the motor output during ongoing locomotion, often referred to as a “Central Pattern Generator” or CPG^21,22^. Indeed, pharmacological activation of the lumbar enlargement, where the hindlimb CPG circuit is thought to reside, can upregulate the frequency of respiratory-like activities on ex vivo preparations from neonatal rats^23,24^. While this is suggestive of ascending projections to respiratory centers, the underlying circuit and its functionality during running has not been documented.

Another gap of knowledge concerns the identification of the respiratory neurons targeted by descending (e.g., from the MLR) or ascending (e.g., from the CPG) locomotor drives. In mammals, the respiratory rhythm is driven by neurons of the pre-Bötzinger complex (preBötC) in the ventromedial medulla^25,26^. The preBötC receives inputs from several brain areas including the midbrain^27^ and, in the lamprey, MLR neurons were shown to contact a presumed homolog of the preBötC^20,28^. This makes the preBötC a prime candidate for promptly entraining respiration during exercise in mammals. More rostrally, the parafacial (pF) respiratory region may be another contender for respiratory regulation during metabolic challenges including effort. In this region, non-catecholaminergic *Phox2b*-expressing neurons (defining the retrotrapezoid nucleus, RTN) can rapidly upregulate respiratory rate in the context of central CO_2_ chemoception^29,30^ and might support active expiration which is thought to accompany exercise^31–35^. *Phox2b-*positive neurons in the pF region have been shown to be activated during running^36^, during locomotor-like activity on ex vivo neonatal rat preparations^23^, and their silencing limits exercise capacity in running rats^37^.

Here we sought to investigate the central circuits interfacing locomotor and respiratory centers in the resourceful mouse model. We found the existence of both a descending drive from the MLR, and of an ascending drive from the locomotor CPG of the lumbar spinal cord. Remarkably, the MLR is capable of upregulating breathing rate even before the initiation of actual limb movements. We further uncovered that the two systems both have access to respiratory rhythm generation mechanisms albeit through two different synaptic schemes. The MLR directly projects to the preBötC, but not to the pF region, while the lumbar spinal cord targets the pF region which in turns contacts the preBötC. Our work therefore demonstrates two locomotor central drives that may underlie breathing adaptability during running and their synaptic nodes in the respiratory central network.

## Results

### Glutamatergic CnF neurons project to the preBötC

**Fig. 1:**
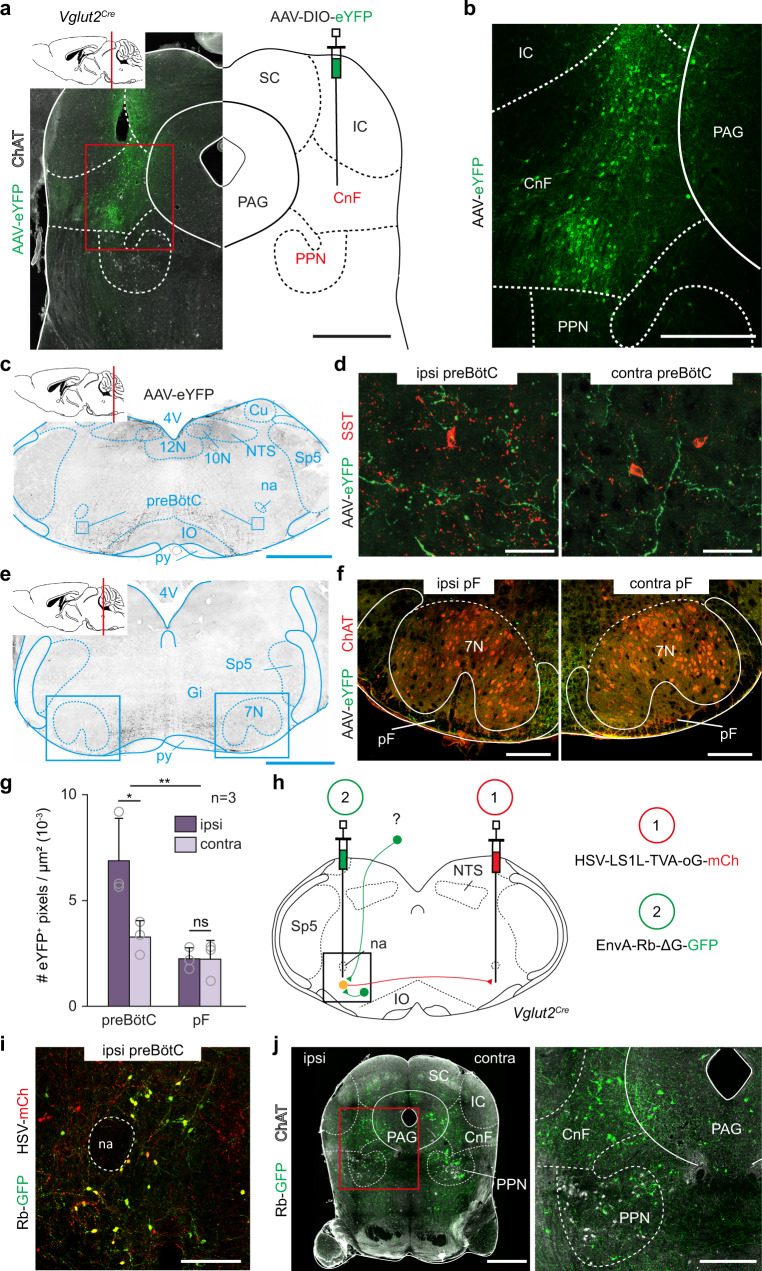
CnF glutamatergic neurons contact the preBötC inspiratory generator. **a** Strategy for tracing glutamatergic (Glut^+^) CnF projections. Cholinergic Pedunculopontine (PPN) neurons are identified by Choline Acetyl Transferase (ChAT) expression. Scale bar, 1 mm. **b** Magnification showing transfected cells in the Cuneiform Nucleus (CnF). Scale bar, 400 µm. **c** Transverse section showing CnF projections in the ventral reticular formation including the preBötC. Scale bar, 1 mm. **d** Magnifications over ipsilateral and contralateral preBötC containing somatostatin-expressing (SST^+^) cells. Note that the CnF projects to both sides with an ipsilateral predominance. Scale bar, 40 µm. **e** Transverse section showing the distribution of CnF projections at the level of the pF area. Scale bar, 1 mm. **f** Magnifications over ipsilateral and contralateral pF areas delineated around ChAT^+^ facial motoneurons (7N). Note very little CnF projections. Scale bar, 250 µm. **a**–**f** are representative of n = 3 animals. **g** Mean density ± SD of eYFP^+^ pixels in the preBötC and the pF areas. Gray circles are mean values of n individual mice. **p = 0.001; *p = 0.011; ns, p = 0.9102 (Wilcoxon matched-pairs tests; preBötC and pF: 9 sections per side). Source data are provided as a Source data file. **h** Strategy for retrograde transsynaptic monosynaptic tracing from Glut^+^ commissural preBötC neurons. EnvA-ΔG-Rb-GFP: G-deleted and EnvA pseudotyped rabies virus. **i** Magnification showing double-transfected “starter” cells in the preBötC. Scale bar, 200 µm. **j** Left: transverse section showing putative presynaptic cells in the CnF and PPN. Scale bar, 1 mm (left), 250 µm (right). **i**, **j** are representative of n = 3 animals (3 males). See also Figs. S1 and S2. Abbreviations used in all figures: PAG periaqueductal gray, IC inferior colliculus, SC superior colliculus, PPN pedunculopontine nucleus, CnF cuneiform nucleus, 4V fourth ventricle, 10N dorsal motor nucleus of vagus, 12N hypoglossal motor nucleus, NTS nucleus tractus solitarius, py pyramidal tract, IO inferior olive, Cu cuneate nucleus, na nucleus ambiguus, preBötC pre-Bötzinger complex, pF parafacial respiratory area, Sp5 spinal trigeminal nucleus, 7 N facial nucleus, Gi gigantocellular reticular nucleus.

We first examined whether locomotor-promoting MLR neurons in mice contact neuronal groups involved in respiratory rhythm generation. The MLR contains two major subdivisions, the cuneiform nucleus (CnF) containing glutamatergic (Glut^+^) neurons, and the pedunculopontine nucleus (PPN) containing both Glut^+^ and cholinergic neurons^15–17^. Since locomotor initiation is attributed to the former, we traced the projections of CnF neurons by unilateral stereotaxic injections of a Cre-dependent AAV-eYFP in *Vglut2*^*Cre*^ adult mice^38^ (Fig. 1a, b, see Fig. S1 for all injection sites). Abundant eYFP-positive fibers were detected in the preBötC, located ventrally to the nucleus ambiguus and containing SST-positive neurons^39^. These projections were found bilaterally with an ipsilateral predominance (Fig. 1c, d, g). In contrast, projections were very sparse in the pF respiratory area, located immediately ventral, ventro-median and ventro-lateral to the facial motor nucleus (7N, Fig. 1e–g). To verify that CnF neurons synaptically target preBötC neurons, we made use of a genetically restricted two-virus approach^40^ to reveal preBötC putative inputs anatomically. A critical population of PreBötC neurons for respiratory rhythm generation are Glut^+^ neurons with commissural projections^41,42^. We therefore injected a retrograde Cre-dependent rabies helper virus (HSV-LSL-TVA-oG^43^) in the preBötC on one side followed by an EnvA-ΔG rabies (Rb) virus in the contralateral preBötC (Fig. 1h). As demonstrated previously^44^, this leads to the expression of the Rb virus in projection-defined neurons (here commissural Glut^+^ preBötC neurons, Fig. 1i) and in their presynaptic partners by transsynaptic spread. The latter were detected in the CnF and PPN nuclei bilaterally (Fig. 1j), as well as on the contralateral preBötC, the NTS, but only few were detected in the pF region (Fig. S2). Altogether, these anterograde and retrograde tracings demonstrate that Glut^+^ CnF neurons directly contact candidate respiratory rhythm generating neurons in the preBötC, but not in the pF region.

### Glutamatergic CnF neurons modulate inspiratory rhythm generation

**Fig. 2:**
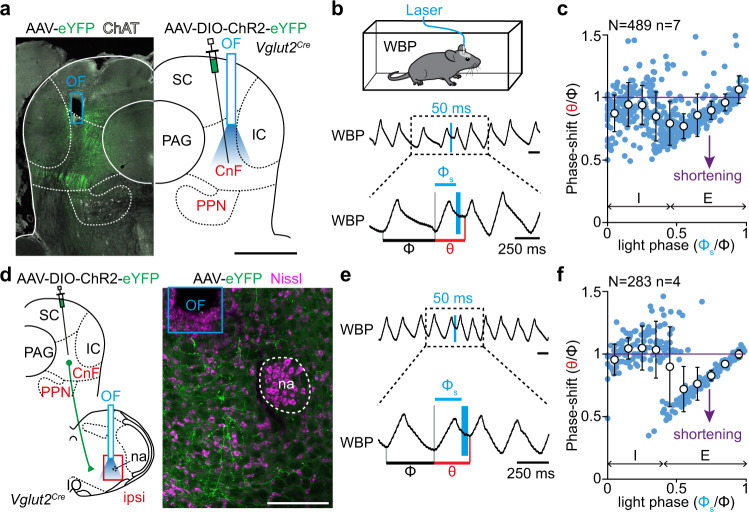
Photoactivation of glutamatergic CnF neurons impacts respiratory rhythm generation in the preBötC. **a** Transverse section showing the experimental strategy for photostimulating Glut^+^ CnF neurons in *Vglut2*^*Cre*^ adult mice. ChAT, Choline Acetyl Transferase. Scale bar, 1 mm. Representative of n = 7 mice. **b** Top: setup for recording ventilation using whole-body plethysmography (WBP) during CnF optogenetic activations. Middle: WBP recordings around a single 50 ms light pulse (vertical blue bar) of Glut^+^ CnF neurons during the expiratory phase of one respiratory cycle (inspirations are upwards, expirations are downwards). Note that the stimulation shortens the respiratory cycle. Bottom: magnification showing the control cycle (φ, black), the phase of light-simulation (φ_s_, blue), and the perturbed cycle (θ, red). **c** Plot of the phase-shift (perturbed cycle normalized to control cycle: θ/φ) as a function of the phase of light-stimulation normalized to the control cycle (φ_s_/φ). Values < 1 (purple line) indicate a shortening of the perturbed cycle. Inspiration (I) and expiration (E) mean durations are indicated. Note that the phase-shift is shortened when the light pulse occurs during expiration. Blue circles represent individual data from N random trials from n mice. White circles are averages ± SD across all trials within 0.1 ms bins. Source data are provided as a Source data file. **d** Left: experimental strategy for photostimulation of Glut^+^ CnF fibers in the ipsilateral preBötC in *Vglut2* ^*Cre*^ adult mice. Right: transverse section at the level of the preBötC showing CnF eYFP^+^ projections and optic fiber placement in the preBötC. Scale bar, 200 µm. Representative of n = 4 mice. **e**, **f** Same as in **b**, **c** during a single 50 ms light-stimulation of CnF projections to the ipsilateral preBötC. Note that the respiratory cycle is shortened when light is delivered during expiration. N random trials from n mice. See also Figs. S3, S4, and S5. Source data are provided as a Source data file.

We next tested the ability of Glut^+^ CnF neurons to functionally impact the preBötC. One hallmark of preBötC responses to phasic incoming inputs is their phase-dependency to the ongoing rhythm^45,46^. To evaluate this for CnF-evoked preBötC responses, we virally delivered the excitatory Channelrhodopsin 2 (ChR2) in Glut^+^ CnF neurons on one side of *Vglut2*^*Cre*^ adult mice, and implanted an optic fiber over the injection site (Fig. 2a). Breathing cycles were measured with whole body plethysmography (WBP, Fig. 2b) while single-pulse (50 ms) photoactivations were delivered randomly during the respiratory cycle during quiet breathing in awake and alert animals. Photostimuli did not elicit any noticeable body movements but indeed evoked individual breaths. We calculated the resultant phase-shift, expressed as the perturbed cycle duration over the duration of the control cycle as done previously^45,46^ (Fig. 2b, see “Methods”). We found that unilateral photostimulation of Glut^+^ CnF neurons elicited an ectopic inspiratory burst and shortened the respiratory cycle but that this effect was most prominent when delivering light-pulses during early expiration (phase: 0.5–0.6, phase shift: 0.77 ± 0.10, p < 0.0001, Fig. 2c). In contrast, the effect was only minimal in late expiration. We also observed a shortening (although less drastic) of the next two subsequent cycles, suggesting that activation of the CnF also determines a longer lasting modulatory action on preBötC rhythm generation (Fig. S3a–d). No alteration of the respiratory cycle was however seen in mock trials in control mice that do not express ChR2 (Fig. S4a, b). Likewise, activating Glut^+^ neurons of the vlPAG, located laterally to the CnF but at the same dorso-ventral depth (Fig. S5a–c), or those of the inferior colliculus, located dorsally but at the same medio-lateral location as the CnF (Fig. S5e–g) did not impact the respiratory cycle. Therefore, the phase-dependent shortening observed when targeting the CnF cannot be attributed to thermal confounds^47^ nor to the few cells in adjacent structures that are occasionally transfected (Fig. S1). To ascertain that this CnF-driven modulation of respiratory rhythm generation owes to direct projections to the preBötC, we next photoactivated ChR2-expressing fibers in the preBötC following viral injection in the CnF (Fig. 2d). This led to similar phase-dependent shortenings of the respiratory cycle, again maximal when light-activations are delivered in early expiration (phase: 0.5 − 0.6; phase shift: 0.79 ± 0.15, p < 0.0001, Fig. 2e, f). This effect disappeared after the second subsequent respiratory cycle (Fig. S3e–h). Light deliveries in the preBötC of control mice that do not express ChR2 did not produce any noticeable effect (Fig. S4d, e). Overall, the direct projections of the Glut^+^ CnF neurons indeed conform to phase-dependent activation of preBötC neurons, highlighting excitatory modulations of inspiratory burst generation.

### Glutamatergic CnF neurons modulate breathing in synergy with locomotion

**Fig. 3:**
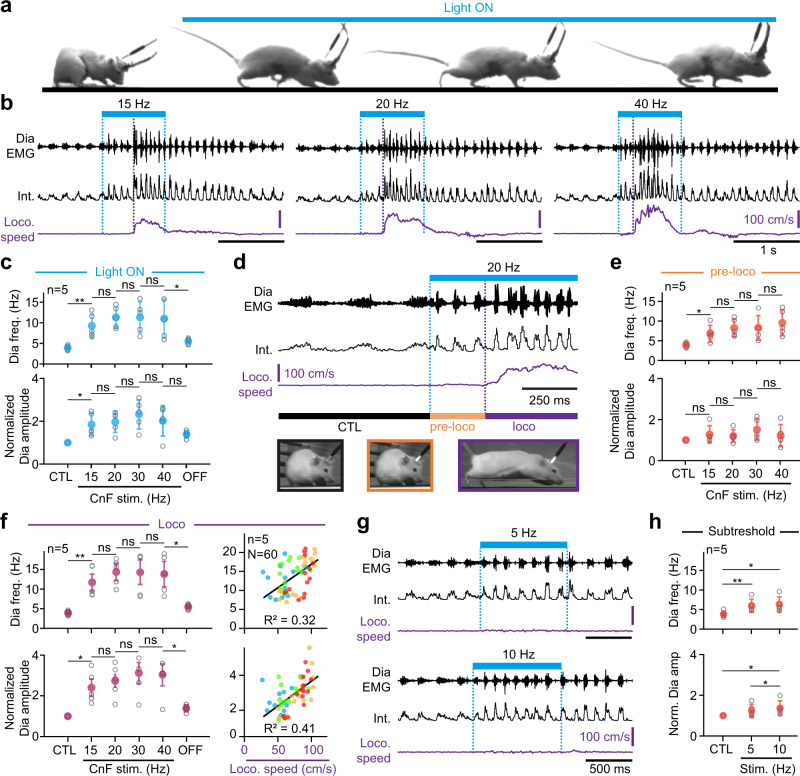
Glutamatergic CnF neurons upregulate breathing in synergy with, and even in the absence of, locomotion. **a** Four representative video snapshots taken before and during the photostimulation of Glut^+^ CnF neurons for 1 s, which triggers running. The experimental strategy is similar to Fig. 2a. **b** Raw (DiaEMG) and integrated (Int.) diaphragmatic electromyogram and locomotor speed during photostimulations. **c** Diaphragmatic frequency and amplitude at rest (CTL), during CnF photostimulation at increasing frequencies, and following light offset (OFF). Amplitudes are normalized to CTL. From left to right: frequency, p = 0,007; p = 0,1046; p = 0,9782; p = 0,7305; p = 0,0392; amplitude, p = 0,0229; p = 0,4756; p = 0,074; p = 0,2066; p = 0,0739. **d** Enlarged EMG recording showing the “pre-loco” and the “loco” phases. **e** Diaphragmatic frequency and amplitude in CTL and during the “pre-loco” phase at increasing CnF photostimulation frequencies. From left to right: frequency, p = 0,0156; p = 0,1128; p = 0,9795; p = 0,1639; amplitude, p = 0,3377; p = 0,108; p = 0,3298; p = 0,5486. **f** Left: similar quantifications during the “loco” phase. From left to right: frequency, p = 0,0067; p = 0,1021; p = 0,9477; p = 0,6789; amplitude, p = 0,0156; p = 0,2892; p = 0,0747; p = 0,6608. Right: diaphragm frequency and amplitude in relation to locomotor speed. Note that 32% of frequency and 41% of amplitude correlate with locomotor speed (*R*^2^). CnF photostimulation frequencies are also indicated by the colored circles (blue: 15 Hz, green: 20 Hz, orange: 30 Hz, Red: 40 Hz). **g** Representative EMG recordings during subthreshold CnF photostimulations that do not initiate running but upregulate breathing frequency and amplitude. **h** Quantifications of diaphragm frequency and amplitude in CTL and during subthreshold CnF stimulations. Frequency: CTL vs 5 Hz, p = 0,00895; 5 Hz vs 10 Hz, p = 0,0618; CTL vs 10 Hz, p = 0,0109. Amplitude: CTL vs 5 Hz, p = 0,148; 5 Hz vs 10 Hz, p = 0,047; CTL vs 10 Hz, p = 0,0746. In graphs, gray circles are means of 3 trials per animal, colored circles are means ± SD across n mice, * denotes p < 0,05, ** denotes p < 0,01, and ns denotes p < 0,05. All tests are matched-pairs *t* tests. For all graphs, source data are provided as a Source data file. See also Figs. S1, S4, S5, S6, and S7.

Through their access to rhythm generating mechanisms in the preBötC, Glut^+^ CnF neurons might be capable of upregulating breathing frequency in synergy with locomotor initiation. To access respiratory parameters during vigorous displacement movements, we made use of our recently-developed method for chronic electromyographic recordings of the diaphragm (DiaEMG), the main inspiratory muscle^4^. *Vglut2*^*Cre*^ animals were made to express ChR2 in Glut^+^ CnF neurons as above, EMG-implanted, and placed in a linear corridor. Light was delivered in 1 s trains at increasing pulse frequencies when animals were quietly breathing, alert, and stationary at one end of the corridor (Fig. 3). Animals were filmed from the side and their displacement speed computed using markerless video-tracking as performed previously^4,48^. In line with numerous studies^15–18^, we found that photoactivating Glut^+^ CnF neurons at 15 Hz or more engages animals in forward locomotion (Fig. 3a, b), and that higher stimulation frequencies impose faster regimes, shorten the delay between light onset and locomotor initiation and increase the occurrence of left-right synchronous gaits (Fig. S6). Our photoactivations did not induce any rearing, grooming, handling behaviors or arrest behaviors that would be expected from light accessing Glut^+^ PPN neurons immediately ventral to the CnF^15,49^. Furthermore, we never observed defensive freezing or orienting behaviors that are typical of activations of, respectively, the adjacent ventrolateral periaqueductal gray (vlPAG^50^, Fig. S5a, b) or the superior or inferior colliculus (refs. ^51,52^, Fig. S5e, f). This, together with the post hoc confirmation of optic fiber placement (Fig. S1), argues for accurately restricted activations of the CnF. Importantly, CnF photostimulations were associated with an increased respiratory rate (Fig. 3b, c), an effect that was not seen in control mice that do not express ChR2 (Fig. S4c). Remarkably, during CnF photoactivations that effectively engage running, respiratory rate increased in a two-step sequential manner. In a first step, that we term the “pre-loco” phase, a modest increase was seen immediately at light onset but before the first locomotor movements (i.e., during the delay between light onset and the initiation of locomotion, Fig. 3d). The mean respiratory rate during this “pre-loco” phase was significantly higher than baseline but not correlated to the photostimulation frequency (Fig. 3e). In a second step, when the animals effectively engage in locomotion (“loco” phase), the respiratory rate was further augmented (Fig. 3d). There, respiratory rate was still not strongly dependent on stimulation frequency but it was proportional to the actual displacement speed (Fig. 3f). The amplitude of DiaEMG was not significantly changed during the pre-loco phase but was increased during the loco phase (Fig. 3e, f), supporting a further ventilatory effort when animals run. We also found that the respiratory and locomotor rhythms were never temporally synchronized (Fig. S7), similarly to the situation in spontaneously running mice^4^. These results indicate that the respiratory frequency during CnF-evoked locomotion is upregulated immediately at light onset before the initiation of locomotion, and further upregulated during actual locomotion. The former observation suggests that Glut^+^ CnF neurons can independently modulate respiratory activity and limb movements. Demonstrating this further, photostimulations below the threshold for locomotor initiation (5 and 10 Hz, Figs. 3g and S6b) were sufficient to significantly increase the respiratory frequency and the amplitude of DiaEMG from baseline (Fig. 3h). The same subthreshold stimulations targeted to the vlPAG or to the inferior colliculus neurons located in proximity to the CnF however evoked no significant changes in breathing rate (Fig. S5d, h), further excluding a confounding effect of the occasional viral expression in these structures.

Altogether, these analyses demonstrate that (i) Glut^+^ CnF neurons can upregulate breathing rate before, or even in the absence of, locomotor movements, (ii) during CnF-evoked locomotion, the highest increase in breathing rate from rest occurs when actual locomotor movements are engaged, (iii) respiratory frequency increase during the “loco” phase is proportional to the displacement speed, and (iv) breaths are not phase-locked to cyclic limb movements.

### The spinal locomotor circuits project to the pF respiratory region

**Fig. 4:**
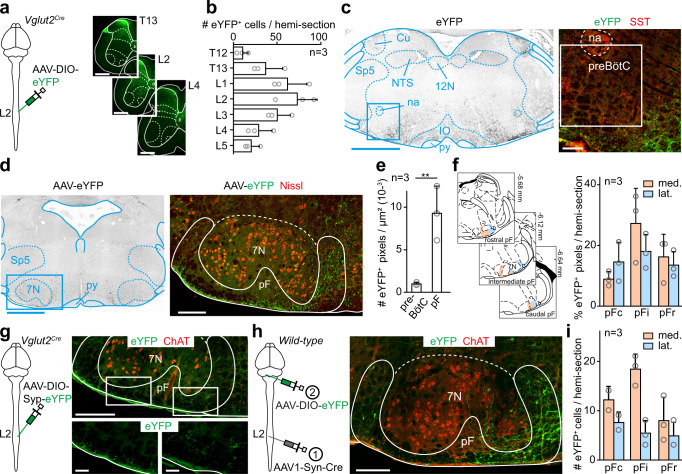
Glutamatergic lumbar spinal cord neurons contact the pF respiratory area. **a** Left: experimental strategy for labeling projections of Glut^+^ thoraco-lumbar neurons bilaterally. Right: transverse sections showing transfected spinal neurons. Scale bars, 500 µm. **b** Distribution of eYFP^+^ transfected cells in different thoraco-lumbar segments. Bar-graphs are the mean number ± SD per hemi-section, and gray circles are mean values of *n* individual mice. **c** Left: transverse section at the preBötC level showing eYFP^+^ spinal projections. Scale bar, 1 mm. Right: magnification of the preBötC area containing SST^+^ cells. Note the absence of spinal projections. Scale bar, 100 µm. Representative of n = 3 mice. **d** Transverse section at the level of the pF showing eYFP^+^ spinal projections. Scale bar, 1 mm. Right: magnification showing abundant eYFP^+^ spinal projections in the pF area, ventrally and ventro-medially to the 7 N motoneurons. **e** Mean density ± SD across n mice of eYFP^+^ fluorescent pixels in preBötC and pF hemi-sections. Gray circles are the mean values of individual mice. ** denotes p = 0.0039 (Wilcoxon matched-pairs tests; 9 sections per area). **f** Left: delineation of rostro-caudal (top to bottom) and medio-lateral (orange and blue) subdivisions of the pF. Right: distribution of fluorescent pixels in the pF subdivisions. Bars are means ± SD across n mice and gray circles are mean values of individual mice. **g** Left: experimental strategy for labeling synaptic boutons of Glut^+^ thoraco-lumbar neurons. Injections were done bilaterally. Right: transverse section showing putative synaptic boutons in the pF area. Scale bars, 250 (top) and 50 (bottom) µm. Representative of n = 3 mice. **h** Left: experimental strategy for labeling pF neurons contacted by spinal neurons through anterograde transsynaptic transfer of an AAV1-Syn-Cre in adult *wild-type* mice. Injections were done bilaterally. Right: transverse section showing eYFP^+^ neurons in the pF area demonstrating that spinal ascending projections make synaptic contacts with pF neurons. Representative of n = 3 mice. Scale bar, 200 µm. **i** Quantification of transsynaptically labeled neurons in the pF subdivisions defined in **f**. See “Methods” for details. For all graphs, source data are provided as a Source data file. See also Fig. S8.

We then reasoned that the engagement in actual locomotor movements may be associated with a stronger drive onto respiratory centers which could originate from activated lumbar locomotor circuits. We thus performed large-volume injections of a Cre-dependent AAV-eYFP bilaterally in the lumbar spinal cord of *Vglut2* ^*Cre*^ mice, to cover most of the thoraco-lumbar enlargement where neurons that generate the locomotor rhythm and set its speed are distributed^53–55^ (Fig. 4a, b), and examined projections in the brainstem respiratory regions. In contrast to the anterograde tracings from the CnF, this revealed very few, if any, eYFP-positive fibers in the preBötC but their abundant (and ubiquitous) presence flanking all but dorsal margins of the 7 N (ventral, anterior, posterior, medial, and lateral, Fig. 4c–f), thus delineating the pF respiratory region in the broad sense (i.e., encompassing subregions involved in chemoception, and in inspiratory and expiratory control^26,35^). To discriminate passing fibers from putative synaptic contacts, we first repeated these spinal cord injections with an AAV that drives expression of a presynaptic, synaptophysin-fused fluorophore^56^, and indeed observed fluorescent puncta in the pF (Fig. 4g). We also performed similar spinal injections using an AAV vector that enables transsynaptic Cre expression in postsynaptic target neurons (AAV1-Syn-Cre^57^) followed by a Cre-dependent AAV-eYFP vector in the pF region. This led to numerous eYFP-expressing cells (Fig. 4h, i) in all subdivisions of the pF. Since the pF does not project to the lumbar spinal cord (Fig. S8), the Cre-dependent labeling is not brought about by retrograde transport of the AAV1-Syn-Cre virus but witnesses its anterograde synaptic transfer. Therefore, ascending spinal projections synaptically target the pF respiratory region.

### Lumbar locomotor circuits upregulate breathing rate through the RTN^*Phox2b/Atoh1*^ ex vivo

**Fig. 5:**
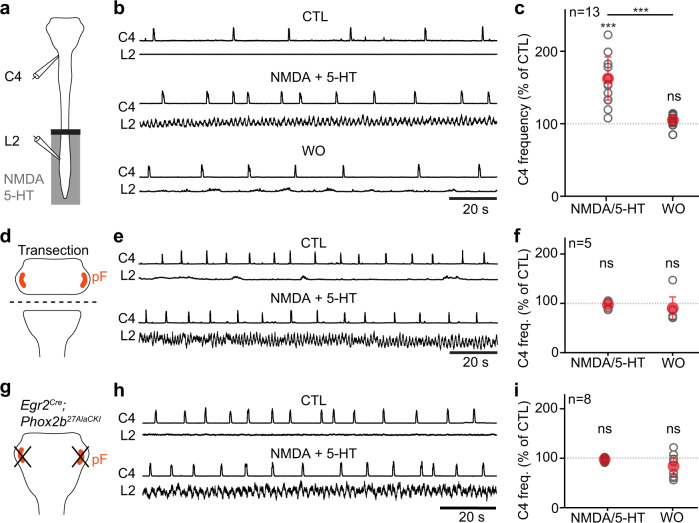
Pharmacological activation of locomotor-like activities increases inspiratory-like frequencies ex vivo, which requires *RTN*^P*hox2b/Atoh1*^ integrity. **a** Experimental strategy for recording respiratory- (C4) and locomotor-like (L2) motor activities on isolated ex vivo brainstem-spinal cord neonatal preparations. The lumbar spinal cord compartment is superfused with control or locomotor drugs enriched (NDMA and 5-HT) aCSF while the brainstem compartment remains in control aCSF. **b** Recordings of C4 and L2 integrated activities of one representative preparation before (CTL), during (NMDA/5-HT), and after (WO) perfusion of locomotor drugs in the lumbar spinal cord compartment. Note that C4 frequency increases when locomotor-like activities are triggered. **c** Quantification of the C4 frequency change (percent change to CTL) during and after drug-induced locomotor-like activity. NMDA/5-HT, p = 0,0002; WO, p = 0,0803; NMDA/5-HT vs WO, p = 0,0002; Wilcoxon matched-pairs tests. **d**–**f** Similar experiments as in (**a-c**) in preparations that underwent a brainstem transection to physically remove the pF respiratory area. No change of respiratory-like activity is seen during the application of locomotor drugs on the lumbar compartment. NMDA/5-HT, p = 0,8125; WO, p = 0,625; Wilcoxon matched-pairs tests. **g**–**i** Similar experiments as in **a**–**c** performed in *Egr2* ^*Cre*^*;Phox2b* ^*27AlaCKI*^ neonates. These lack pF neurons that express *Phox2b* and are derived from rhombomere 5, a genetic intersection that recapitulates the genetically-defined RTN^*Phox2b/Atoh1*^. No change of respiratory-like activity is seen during the application of locomotor drugs on the lumbar compartment. NMDA/5-HT, p = 0,1094; WO, p = 0,1953 ; Wilcoxon matched-pairs tests. For all graphs, gray open circles are means of individual preparations, red circles are means ± SD across n preparations, *** denotes p < 0,001, and ns denotes p > 0,05. Source data are provided as a Source data file. See also Figs. S9 and S10.

To investigate functionally the possibility that lumbar locomotor circuits can upregulate breathing, we used ex vivo isolated brainstem/spinal cord preparations from neonatal mice. Although long-used for monitoring locomotor^55^ or respiratory-like^25^ activities, monitoring both simultaneously during drug-evoked locomotor-like activity had only been achieved on rat preparations^23,24^. We adapted the method to the neonatal mouse and used a split-bath for independent pharmacological manipulation of the brainstem and spinal cord through superfusion of dedicated artificial-cerebrospinal fluids (aCSFs, see “Methods”). In these conditions, we recorded respiratory-like activity on the 4^th^ cervical ventral root and locomotor-like activity on the 2^nd^ lumbar ventral root (Fig. 5a).

When both the brainstem and spinal cord were superfused with control aCSF, only respiratory-like activities were detected. Bath-application of the neuroactive substances *N*-methyl-d-aspartate (NMDA) and serotonin (5-HT) in the spinal compartment evoked locomotor-like activities associated with an increased frequency of the respiratory-like activity (by 162 ± 30% of baseline, Fig. 5b, c). To rule out drug leakage from the spinal compartment, we first verified that this frequency increase of respiratory-like activities was abolished following a cervical transection (Fig. S9a–c). We also found a similar acceleration of respiratory-like activity by targeted optogenetic activations of lumbar Glut^+^ neurons (170 ± 38% from baseline, Fig. S9d–f). Altogether, these experiments indicate that the activation of lumbar spinal circuits that contain the locomotor CPG exerts an excitatory effect on respiratory activity.

Since our anatomical observations place the pF respiratory region as a candidate target of ascending pathways from the spinal cord (Fig. 4), we next addressed the functional contribution of this region. We first eliminated physically the pF by a complete transection below the facial motor nucleus (Fig. 5d). Inspiratory-like activities persisted on the 4^th^ cervical root as expected^58^, but pharmacological activation of lumbar circuits no longer significantly upregulated their frequency (Fig. 5e, f). We also examined specifically the contribution of RTN neurons in the pF region^29,30^, making use of the fact that RTN neurons relevant for modulating respiratory rhythm generation, at least in the context of central chemoception, (i) are best identified by a combined history of expression of the transcription factors *Phox2b* and *Atoh1*^59,60^ (thereafter RTN^*Phox2b/Atoh1*^ neurons); and (ii) can be deleted when expressing a mutated allele of PHOX2B (*Phox2b*^*27Ala*^) in rhombomeres 3 and 5 (*Egr2* ^*cre*^*;Phox2b* ^*27AlaCKI*^ background^59,60^; Fig. S10). We thus recorded respiratory- and locomotor-like activities from *Egr2* ^*cre*^*;Phox2b* ^*27AlaCKI*^ RTN mutant pups (Fig. 5g). Preparations showed persistent inspiratory-like activity on the 4^th^ cervical root, but pharmacological activation of lumbar circuits no longer upregulated its frequency (Fig. 5h, i). These experiments highlight the capacity of spinal lumbar circuits to upregulate respiratory-like activities through RTN^*Phox2b/Atoh1*^ neurons in the pF respiratory region.

### Silencing RTN^*Phox2b/Atoh1*^ neurons reduces respiratory increase during running exercise in vivo

**Fig. 6:**
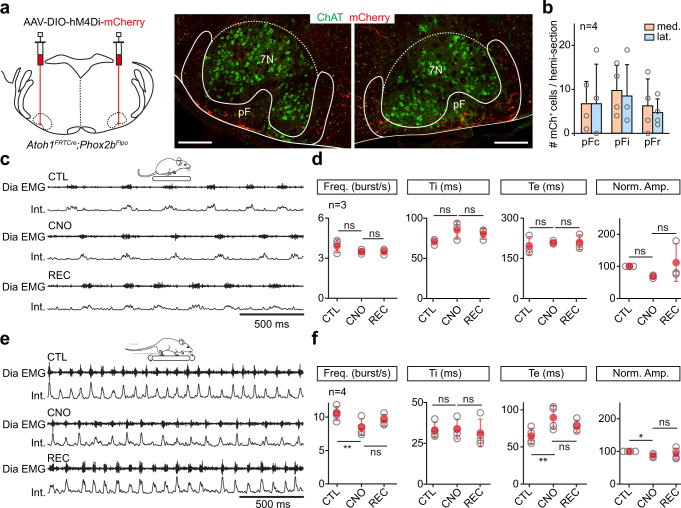
Silencing RTN^*Phox2b/Atoh1*^ neurons reduces respiratory frequency during running. **a** Left: experimental strategy for silencing RTN^*Phox2b/Atoh1*^ neurons bilaterally using the virally-driven inhibitory DREADD receptor hM4Di in *Atoh1*^*FRTCre*^*;Phox2b*^*Flpo*^ adult mice. Right: transverse sections showing bilaterally transfected neurons in the pF area around the 7N motoneurons identified by Choline Acetyl Transferase (ChAT) expression. Scale bar, 250 µm. Representative of n = 4 mice. **b** Number and distribution of transfected (mCherry^+^) neurons across pF subregions as defined in Fig. 4f. Bars are means ± SD across n mice and gray circles are mean values of individual mice. See methods for details. From left to right: frequency: p = 0,2532; p > 0,9999; Ti: p = 0,1975; p = 0,6264; Te: p = 0,6042; p = 0,998; amplitude: p = 0,1716; p = 0,1975 (Wilcoxon matched-pairs tests). **c** Raw (DiaEMG) and integrated (Int.) recordings of diaphragm activity of one representative animal at rest before (CTL), during (CNO), and after (REC) administration of CNO at 10 mg/kg. **d** Quantification of respiratory parameters before, during and after CNO administration: frequency, inspiratory (Ti) and expiratory (Te) times, and normalized amplitude. Note that silencing RTN^*Phox2b/Atoh1*^ neurons does not alter basal respiratory parameters. From left to right: frequency: p = 0,2532; p > 0,9999; Ti: p = 0,1975; p = 0,6264; Te: p = 0,6042; p = 0,998; amplitude: p = 0,1716; p = 0,1975 (Wilcoxon matched-pairs tests). **e**, **f** Similar representations as in **c**, **d** in mice running on a treadmill at 40 cm/s. Note the significantly lower diaphragm frequency and increased expiratory time (Te) when RTN^*Phox2b/Atoh1*^ neurons are silenced. From left to right: frequency: p = 0,0069; p = 0,1025; Ti: p = 0,5311; p = 0,3134; Te: p = 0,0089; p = 0,0987; amplitude: p = 0,0146, p = 0,4805 (paired *t* tests). For all graphs, gray open circles are means of individual mice, red circles are means ± SD across n mice, * denotes p < 0,05, ** denotes p < 0,01, *** denotes p < 0,001, and ns denotes p > 0,05. Source data are provided as a Source data file. See also Fig. S11.

The importance of RTN^*Phox2b/Atoh1*^ neurons revealed above ex vivo prompted us to address their contribution to respiratory activity during running in behaving mice. For this, we used an intersectional background in which Cre expression is conditioned by both *Atoh1* and *Phox2b* expression^60^ (*Atoh1*^*FRTCre*^*;Phox2b*^*Flpo*^), and injected in the pF region bilaterally a Cre-dependent AAV coding the inhibitory DREADD receptor hM4Di (Fig. 6a, b). Diaphragmatic EMG electrodes^4^ were then implanted and respiratory parameters were measured before and 2-3 h after the administration of the DREADD ligand Clozapine-*N*-oxide (CNO) at rest or during treadmill running. At rest, CNO administration had no significant effect on the mean inspiratory frequency, inspiratory and expiratory durations, DiaEMG amplitude (Fig. 6c, d) and breathing variability (coefficient of variation <0.1), supporting previous findings that the RTN only minimally contributes to the baseline breathing rate^37^. When animals were made to run at 40 cm/s they showed, prior to CNO administration, an augmented respiratory frequency (269% increase) and amplitude (263% increase), in agreement with previous work^4^. However, following CNO administration, their breathing rate and DiaEMG amplitudes were significantly reduced compared to the pre-CNO measurements at the same running speed (Fig. 6e, f). Furthermore, CNO-treated mice showed a less pronounced reduction in their expiratory time (Te). Administration of saline in hM4Di-injected mice, or of CNO on wild-type mice, changed neither the breathing rate, expiratory and inspiratory times, nor the DiaEMG amplitude both at rest and during running sessions (Fig. S11). We also did not observe any impact of CNO or saline alone on breathing regularity (coefficient of variation <0.1). This excludes any non-specific effects of CNO alone, as previously reported for the same concentration^61^. These experiments indicate that the activity of RTN^*Phox2b/Atoh1*^ neurons is required for upregulating ventilatory frequency during running exercise.

### RTN^*Phox2b/Atoh1*^ neurons project to the preBötC inspiratory generator

**Fig. 7:**
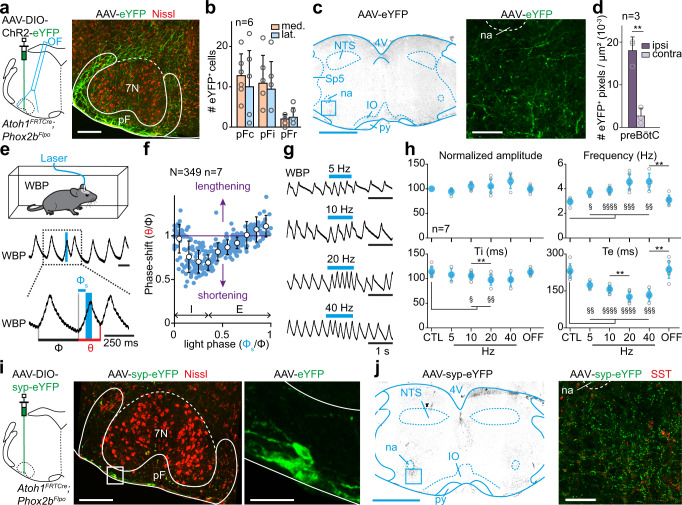
RTN^*Phox2b/Atoh1*^ neurons contact the preBötC and impact respiratory rhythm generation. **a** Left: strategy for labeling and photoactivating RTN^*Phox2b/Atoh1*^ neurons unilaterally. Right: transfected somas. Scale bar, 250 µm. Representative of n = 6 mice. **b** Distribution of transfected neurons across pF subregions defined in Fig. 4f. Bars are means ± SD across n mice and circles are mean values of individual mice. **c** Transverse section at the preBötC level showing RTN^*Phox2b/Atoh1*^ projections. Scale bar, 500 µm (left), 100 µm (right). **d** Distribution of RTN^*Phox2b/Atoh1*^ projections in the preBötC. Bars are means ± SD across n mice and gray circles are mean values of individual mice. **p = 0.0039, Wilcoxon matched-pairs test. **e** Top: whole-body plethysmography recording (WBP, inspirations are upwards) around a single photoactivation of RTN^*Phox2b/Atoh1*^ neurons which shortens the respiratory cycle. Φ: control cycle, φ_s_: phase of photoactivation, θ: perturbed cycle. **f** Inspiratory phase-shift as a function of the phase of photoactivation showing a shortening. Blue circles are N random trials from n mice, white circles are averages ± SD across all trials within 0.1 ms bins. See Fig. 2 and methods for details. **g** WBP recordings during RTN^*Phox2b/Atoh1*^ photoactivation at increasing frequencies. **h** Respiratory parameters before (CTL), during and after (OFF) photoactivations. Gray circles are means of individual animals and colored circles are means ± SD across n mice. In all graphs, §, p < 0.05; ** or §§, p < 0.01, §§§, p < 0.001; §§§§, p < 0.0001 using Wilcoxon matched-pairs test (amplitude) and paired *t* tests (frequency, Ti, Te). Exact p values between conditions (*) are, from left to right: amplitude: p = 0,25; p = 0,9375; p = 0,3125; p = 0,125; frequency: p = 0,125; p = 0,1215; p = 0,3527; p = 0,0066; Ti: p = 0,684; p = 0,0025; p = 0,2645; p = 0,0523; Te: p = 0,071; p = 0,0069; p = 0,1537; p = 0,0014. Additional statistics compared to CTL condition (§): amplitude: CTL vs 5 Hz, p = 0,125; CTL vs 10 Hz, p = 0,2188; CTL vs 20 Hz, p = 0,4688; CTL vs 40 Hz, p = 0,125; CTL vs OFF, p = 0,8125; frequency: CTL vs 5 Hz, p = 0,0204; CTL vs 10 Hz, p < 0,0001; CTL vs 20 Hz, p = 0,0001; CTL vs 40 Hz, p = 0,0017; CTL vs OFF, p = 0,3061; Ti: CTL vs 5 Hz, p = 0,0651; CTL vs 10 Hz, p = 0,042; CTL vs 20 Hz, p = 0,0031; CTL vs 40 Hz, p = 0,0903; CTL vs OFF, p = 0,7401; Te: CTL vs 5 Hz, p = 0,0082; CTL vs 10 Hz, p < 0,0001; CTL vs 20 Hz, p < 0,0001; CTL vs 40 Hz, p = 0,0003; CTL vs OFF, p = 0,6141. **i** Left: strategy for labeling RTN^*Phox2b/Atoh1*^ synaptic contacts unilaterally. Middle: transverse section showing transfected somas in the pF. Scale bar, 250 µm. Right: magnification. Scale bar, 50 µm. **j** Transverse section at the preBötC level showing RTN^*Phox2b/Atoh1*^ synaptic contacts. Scale bar, 1 mm (left), 100 µm (right). **i**, **j** are representative of n = 4 animals. For all graphs, source data are provided as a Source data file. See also Fig. S12.

The reduced breathing rate following the silencing of RTN^*Phox2b/Atoh1*^ neurons suggests that this genetically defined neuronal subset may have access to the main inspiratory generator, the preBötC. While the pF region was shown to send projections to the ventral respiratory column and possibly the preBötC^62,63^, this had not been determined for RTN^*Phox2b/Atoh1*^ neurons. To examine it, similarly to what we did for CnF neurons (Figs. 1 and 2), we injected a Cre-dependent AAV coding ChR2 and a fluorescent protein in the pF region of *Atoh1*^*FRTCre*^*;Phox2b*^*Flpo*^ animals and implanted an optic fiber above the injection site (Fig. 7a, b). Anatomically, we observed and quantified abundant eYFP-labeled varicosities of RTN^*Phox2b/Atoh1*^ neurons in the preBötC region with a strong ipsilateral dominance (Fig. 7c, d). To demonstrate the capacity of RTN^*Phox2b/Atoh1*^ neurons to directly modulate rhythm generation mechanisms in the preBötC, we examined respiratory responses to 50 ms single-pulse photoactivations with the same analytic tools described earlier for CnF activations (Fig. 2). We found that unilateral photostimulation of RTN^*Phox2b/Atoh1*^ neurons could elicit an ectopic inspiratory burst and shorten the respiratory cycle (Fig. 7e, f). However, when compared to CnF photostimulation, shortenings of the respiratory cycle peaked earlier during the inspiration phase (phase 0.2 − 0.3 and 0.3 − 0.4; phase shift: 0.70 ± 0.13 and 0.69 ± 0.09; p < 0.0001) and, contrary to the CnF, stimulations during late expiration caused a significant lengthening of the respiratory cycle (phases 0.9 − 1; phase shift: 1.11 ± 0.12; p = 0.0004). Here again, stimulation of RTN^*Phox2b/Atoh1*^ neurons led to phase dependent inspiratory responses in keeping with the preBötC excitability dynamics. Yet unlike the CnF, no shortening of the subsequent cycles following the stimulus was observed (Fig. S12). Moreover, 1 s light stimulations of the RTN^*Phox2b/Atoh1*^ neurons resulted in significant augmentations of respiratory frequency during the stimulus, mostly accounted for by a decrease of Te, even at the lowest stimulation frequencies (Fig. 7g, h). RTN^*Phox2b/Atoh1*^ to preBötC connectivity is further corroborated by the detection of eYFP puncta in the preBötC following the injection of a Cre-dependent AAV coding a synaptophysin-fused eYFP in the pF area on *Atoh1*^*FRTCre*^*;Phox2b*^*Flpo*^ animals (Fig. 7i, j). Altogether, these results demonstrate that the genetically defined subset of RTN^*Phox2b/Atoh1*^ neurons can upregulate respiratory rate through direct projections to the preBötC.

## Discussion

**Fig. 8:**
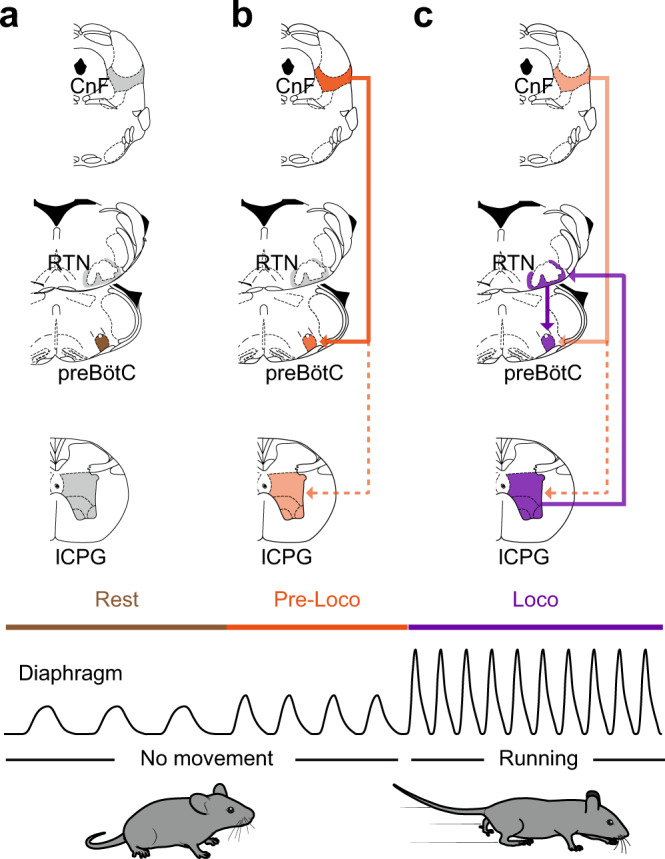
Graphical representation of revealed circuits. **a** At rest, and in the absence of locomotor initiation signal from the CnF to the locomotor central pattern generator (lCPG), the preBötC drives the basal inspiratory rate. **b** CnF activation leads to an augmented ventilation before the animal engages in effective running. This “pre-loco” phase is attributed to the CnF sending a direct and rapid activation signal to the preBötC (thick line). In contrast, the CnF signal crosses multiple synapses before reaching the locomotor CPG^16,65^ (dashed line), which may support a longer latency to locomotor onset. **c** CnF activation eventually leads to a running episode, during which ventilation is augmented further. This effect is attributed to the activated lCPG sending direct projections to the pF respiratory area, and in particular to the RTN^*Phox2b/Atoh1*^ neurons, which in turns contact and activate the preBötC (thick lines).

A neuronal substrate for hyperpnea during running has long been proposed but its underlying cells and circuits had remained speculative. We uncover here two systems by which the central locomotor network can enable respiratory rate augmentation in relation to running activity (Fig. 8). On the one hand, we reveal the capacity of the MLR subnucleus CnF, a conserved locomotor controller^12,15–19^, to upregulate breathing. On the other hand, we demonstrate that when active, the lumbar enlargement of the spinal cord containing the hindlimb CPG, also potently upregulates breathing rate. Using cell-type specific circuit tracing and functional interferences, we further characterize each locomotor drive by identifying its specific neuronal target in the respiratory network.

### Multiple locomotor drives set respiratory rhythm frequency

A remarkable finding reported here is the function of the CnF beyond locomotor control. We should stress again here that our injection scheme only minimally transfects, and likely does not photoactivate, neurons in the adjacent PAG and IC (Fig. S1). While these structures may modulate breathing in specific contexts^64^, they do not contribute to the modulations reported here (Fig. S5). One intriguing feature we observed with CnF stimulations is that respiratory rate is upregulated before the engagement of locomotor movements, or for stimulations that are below the threshold for locomotor initiation (Fig. 3). This might be consequent to the direct access of the CnF to the preBötC (Figs. 1 and 2), while its access to the limb circuits requires the crossing of multiple synapses, including reticulospinal relay neurons^16,65^ (Fig. 8b). The regulation of breathing by the CnF in the absence of locomotor movements places the CnF as a bona fide respiratory modulator structure. Admittedly, we could not assess the necessity of these neurons for the development of the ventilatory response during spontaneous running exercise since their silencing itself impairs the capacity of the animal to actually run, and therefore exercise^16^. Nevertheless, in the lamprey, spontaneous swimming bouts are preceded by a marked increase in respiratory frequency^20,28^. Activity of the CnF prior to movement initiation may hence bear physiological relevance as an anticipatory mechanism to the planned motor action. Human subjects informed of an upcoming exercise or imagining performing an exercise^7–9^ also show increased ventilation and cardiovascular responses, although less drastic than during actual movements. These adjustments are reminiscent of the “pre-loco” phase resulting here from CnF stimulations (Fig. 3). Interestingly, the CnF is associated with escape-like fast regime running^15,16^, and may be part of a larger command system for defensive behaviors in the broad sense^66,67^. It may therefore bear output connectivity allowing to engage a composite response with both cardiovascular^67^, respiratory (this study and ref. ^20^), and if needed, locomotor components. Data in the lamprey indicate that locomotor and respiratory centers are contacted by distinct MLR neurons^20^. Pending an equivalent examination in mice, it is possible that the MLR may, similarly to other descending motor pathways^44^, host projection-defined subsets that each control one trait of a multi-faceted behavior.

We also report the existence of a modulation of breathing rate upon activation of the lumbar spinal segments that contains the hindlimb locomotor circuits^21,22^ (Fig. 8c). Such an ascending drive, previously suggested in rats^23,24^, is demonstrated here in mice by local pharmacological (Fig. 5) or optogenetic (Fig. S9) activations of the lumbar enlargement on reduced preparations ex vivo. Indeed, ex vivo, the absence of the MLR and of peripheral structures as well as the experimental control of the extracellular solution, allow to isolate the functional contribution of the spinal ascending drive from descending, peripheral feedbacks, and central chemoceptive ones. We hence propose that active locomotor executive circuits (through projections to the RTN, see below), continuously inform respiratory centers on the state of ongoing locomotion and are, at least partly, causal to the further increase of respiratory rate seen when animals engage in locomotion (i.e., the “loco” phase) following CnF stimulations (Fig. 8c). This is further supported by the recent report of increased respiratory-like activity after activation of the lumbar enlargement in rat neonatal preparations^23^. Yet, we cannot rule out a persisting activity of CnF neurons during the running exercise^16^. This leaves open the possibility that the two locomotor drives, i.e., from the CnF and the lumbar spinal cord, may synergize to set the respiratory frequency during ongoing running.

### Different respiratory nodes integrate distinct locomotor drives

Another intriguing observation is that the two revealed locomotor drives target different nuclei in the respiratory rhythm generating network. The CnF connects to the preBötC, the main site of inspiratory rhythm generation^25,26^, while the lumbar CPG contacts the pF respiratory region, and possibly the RTN^*Phox2b/Atoh1*^ previously implicated in central CO_2_ chemoception^29,30,59,60^ that in turn projects onto the preBötC (Fig. 8b, c).

For the former, the connectivity is first demonstrated anatomically by the detection of anterogradely labeled fibers, and a transsynaptic labeling approach initiated from Glut^+^ preBötC neurons (Fig. 1). This connectivity is also supported functionally, by the capacity of Glut^+^ CnF neurons or their projections in the preBötC to impact respiratory rhythm mechanisms (Fig. 2). In contrast, we found that ascending projections from the lumbar spinal cord were virtually absent in the preBötC but were dense in the pF (Fig. 4), in an area compatible with that of the RTN. The synaptic nature of these ascending projections was ascertained using a synaptic labeling (Fig. 4g) and an anterograde transsynaptic strategy^57^ (Fig. 4h). The functionality of these ascending projections and the identity of their neuronal targets as RTN^*Phox2b/Atoh1*^ neurons were demonstrated both by silencing experiments in vivo (Fig. 6) and by ex vivo experiments on RTN null mutants (Fig. 5). In the latter situation, peripheral chemoreceptors, sensory activation or other metabolic factors do not contribute. Hence, the spinal locomotor circuits provide at least one source of RTN^*Phox2b/Atoh1*^ neuron excitation, which may be complemented in vivo by other sources including neighboring adrenergic C1 neurons^29,68^ or somatic afferents^69^. The requirement of the RTN is further supported by the perturbed respiratory-like activity following bilateral lesions of the RTN in rat neonatal preparations^23^, and the increased c-Fos expression in RTN neurons following exercise in adult rats^36^. Furthermore, a broader silencing of RTN and neighboring adrenergic neurons, defined by *Phox2b* expression alone, was previously shown to limit the running capacity in rats^37^. Therefore, the RTN^*Phox2b/Atoh1*^ subset may be the prominent integrator of the ascending locomotor drive, at least for the setting of respiratory frequency. A previous study (in rats) of the role of the RTN in mediating changes in the respiratory activity during exercise concluded that the RTN does not appear to be involved in triggering the initial increases in ventilation at the onset of exercise but is rather critical in maintaining increased respiratory effort during sustained exercise^37^. Our work strikingly substantiates this view. The CnF would pre-empt and trigger heightened metabolic demand at the onset of exercise while the RTN, by adjusting the respiratory effort to the engaged and sustained locomotion, might determine exercise capacity.

We also show that the RTN^*Phox2b/Atoh1*^ neurons in turn project to the preBötC (Fig. 7). This makes the preBötC inspiratory generator a final integrator of both the descending (from the CnF, Fig. 8b) and the ascending (from the lumbar spinal cord, Fig. 8c) locomotor drives and raises the question of the identity of the targeted neurons. Our transsynaptic tracing scheme from the preBötC demonstrates that the CnF targets glutamatergic preBötC neurons (Fig. 1h–j). Comparatively however, the number of transsynaptically labeled neurons in the pF area was much lower (Fig. S2d, e). Moreover, brief CnF photostimulations were most effective to shorten the respiratory cycle when delivered during early expiration but failed when delivered during inspiration (Fig. 2). This is reminiscent to what was observed when directly stimulating glutamatergic preBötC neurons collectively^46^ or the *Dbx1*-expressing V0 subset^45^, the main rhythmogenic candidates^41^. In contrast, when stimulating the RTN^*Phox2b/Atoh1*^, the shortening of the respiratory cycle is most efficient during inspiration and a significant lengthening of the respiratory cycle is observed when photoactivations are delivered in late expiration (compare Fig. 2c with Fig. 7f). This permissive action in inspiration and the lengthening in expiration recall what others have reported when specifically activating inhibitory, but not excitatory, preBötC neurons^46^. The RTN^*Phox2b/Atoh1*^ and the CnF might thus preferentially target different cell-types in the preBötC: inhibitory neurons for the former, and glutamatergic ones for the latter. Note that such a bias towards preBötC inhibitory target neurons for the RTN^*Phox2b/Atoh1*^ compared to the CnF would be mechanistically compatible with switching from CnF-induced “pre-loco” moderate respiratory frequency increase, to the higher “loco” respiratory frequency range associated to actual locomotion. Indeed, the current model ascribes a limited ability of preBötC excitatory neurons compared to inhibitory neurons at entraining high frequency rhythms^46^. Although our data are compatible with such a working model, the proposed connectivity will need to be investigated directly by future work.

### Limitations and perspectives

While both the CnF and spinal locomotor circuits have the capacity to upregulate breathing frequency, further adjustments can be achieved by changes in tidal volume and by the mobilization of active expiration, a candidate signature of exercise hyperpnea. The expiratory motor drive was not directly monitored here and recording the activity of abdominal respiratory muscles would be required. The possibility that the spinal ascending locomotor drive contributes to the onset of active expiration is yet likely given that (i) Te was found considerably reduced in ventilatory responses to 1 s photostimulations of RTN^*Phox2b/Atoh1*^ neurons (Fig. 7h) as expected from induction of an active expiratory phase, (ii) the widespread projections onto the pF region, including its lateral aspect (Fig. 4f) thought to host an expiratory oscillator^26,35^, and (iii) the recent demonstration that optogenetic activation of CO_2_ chemoceptive RTN neurons induces active expiration^34^. Finally, while our results localize the locomotor ascending drive to the lumbar spinal cord, the identity of incriminated neurons will remain to be characterized. Our spinal injections were intentionally targeted to the entire thoraco-lumbar enlargement, since the locomotor-promoting circuits are distributed across multiple segments^55^. Spinal neurons of V2a, V0_V_ or V3 genetic identity as well as *Shox2* and *Hb9*-expressing ones stand as candidates, by virtue of their glutamatergic nature and their direct contribution to locomotor rhythm and pattern^21,70^. In any case, should these neurons and possibly the ensuing ascending drive be rhythmically modulated during locomotion, this fails to translate in a phased entrainment of breathing (this study and ref. ^4^).

### General conclusion

We provide here a firm demonstration and a circuit characterization of two central neuronal drives for breathing adaptation during running. The circuit revealed highlights the multi-functional ambition of cell-types and pathways that are typically regarded as “locomotor” or “respiratory” related. This represents an entry point to further decipher the nodes and links through which distinct motor programs necessarily cooperate.

## Methods

### Mice

C57BL/6J wild-type mice were obtained from Janvier Labs (Le Genest-Saint-Isle, France). *VGlut2-IRES-Cre* animals (therefafter *Vglut2*^*Cre*^,^38^ and *Ai32(RCL-ChR2(H134R)/EYFP) (*thereafter *ChR2*^*floxed*^, ref. ^71^) were obtained from Jackson Laboratories. To manipulate RTN^*Phox2b/Atoh1*^ neurons we used the following mouse lines: *Egr2*^*Cre*^^72^ crossed with *Phox2b*^27A*la*CKI59^ and *Atoh1*^*FRTCre*^*; Phox2b*^*Flpo*^^60^ mouse lines. All animals were kept on the C57BL/6J background and group-housed with free access to food and water in controlled temperature (21 °C) and humidity (between 40 and 55%) conditions and exposed to a conventional 12-h light/dark cycle. Experiments were performed on animals of either sex, aged 2 to 3 months at the time of first injection and data from males and females were pooled. The distribution of males and females for all experimental condition is presented in the Source data files. All procedures were approved by the French Ethical Committee (“Comité d’éthique en Expérimentation Animale”, CEEA #59, authorization 2020-022410231878) and conducted in accordance with EU Directive 2010/63/EU. All efforts were made to reduce animal suffering and minimize the number of animals.

### Viruses used

For anterograde tracings and photostimulations of the CnF and its projections in the preBötC, as well as for photoactivations of IC and the PAG, we used a Cre-dependent AAV9-Ef1a-DIO-hChR2(E123T/T159C)-eYFP (Addgene #35509, titer 7.7e12 vp/ml^44,73^) unilaterally (40 to 100 nL). No labeling was seen in wild-type animals (see also ref. ^73^ and^44^ for validation studies). For anterograde tracing from the lumbar spinal cord, the same virus was injected bilaterally (600-750 nL/side) in the second lumbar segment, and for RTN^*Phox2b/Atoh1*^ photostimulations, the injection was unilateral (350-500 nL). For reversible silencing of RTN^*Phox2b/Atoh1*^ neurons, we used bilateral injections (350-500 nL/side) of an AAV8.2-hEF1a-DIO-hM4Di-mCherry-WPRE^44^ obtained from Dr. Rachael Neve (Gene Delivery Technology Core, Massachusetts General Hospital, USA, titer: 5.6e12 vp/ml). No labeling was seen in wild-type mice. For transsynaptic labeling of inputs onto preBötC neurons, we used 500 nL of a HSV1-hEF1a-LS1L-TVA950-T2A-rabiesOG-IRES-mCherry obtained from Dr. Rachael Neve (Gene Delivery Technology Core, Massachusetts General Hospital, USA), and 200 nL of EnvA-∆G-rabies-GFP obtained by the GT3 core (Salk Institute, USA, titer: 9.59e9 vp/ml), with inspiration from previous work^44^. For anterograde transsynaptic tracing, we used injection of an AAV2/1-hSyn-Cre-WPRE-hGH (UPenn Vector Core, titer: 6.68e13 vg/ml, validated in^57^) bilaterally in the lumbar spinal cord (600-750 nL/side) and the same AAV (Addgene # 35509) as for anterograde tracing and optogenetic stimulations bilaterally in the pF area (300 nL/side). For anterograde synaptic tracing from the RTN^*Phox2b/Atoh1*^, we used unilateral injection (350-500 nL) of an AAV8.2-hEF1a-DIO-synaptophysin-eYFP^56^ obtained from Dr. Rachael Neve (Gene Delivery Technology Core, Massachusetts General Hospital, USA, titer: 5.4e12 vp/ml). No labeling was seen in wild-type mice.

### Surgical procedures

#### Injections and implants in the brainstem

Procedures are inspired by previous work^44^. Animals were anesthetized with isoflurane throughout the surgery (4% at 1 L/min for induction; 2-3% at 0.2 L/min for maintenance). Buprenorphine (0,025 mg/kg) was administered subcutaneously for analgesia before the surgery. The temperature of the mice was maintained at 36 °C with a feedback-controlled heating pad. Anesthetized animals were placed on a stereotaxic frame (Kopf) and the skull was exposed. Viral vectors were delivered using a pulled glass pipette connected to a syringe pump (Legato 130, KD Scientific, customized by Phymep, France). Infusion flow was set to 100 nL/min. Coordinates (in mm) used to target CnF neurons (Figs. 1–3) were: 4.4 caudal to bregma, 1.3 lateral, and 2.8 mm from skull surface. Coordinates used to target PAG neurons (Fig. S5) were: 4.4 caudal to bregma, 0.3 lateral, and 2.8 mm from skull surface. To target IC neurons (Fig. S5), they were: 4.4 caudal to bregma, 1.3 lateral, and 1.7 mm from skull surface. In both cases, the volume injected was 50 nL. RTN^*Phox2b/Atoh1*^ neurons were targeted unilaterally (Fig. 7) or bilaterally (Figs. 4 and 6) using the following coordinates: 6.0 caudal to bregma, 1.25 lateral, and 5.8 from skull surface. preBötC injections (Fig. 1) were performed at the following coordinates: −7.2 from bregma, 1.25 lateral, and 5.7 from skull surface. After the injection, the pipette was held in place for 5 to 10 min before being slowly retracted. For CnF, CnF fibers in the preBötC, RTN^*Phox2b/Atoh1*^, PAG and IC optogenetic activations, a 200 µm core 0.39 NA optic fiber connected to a 1.25 mm diameter ferrule (Thorlabs) was implanted 0.4 mm above targeted sites. Optic fibers were secured to the skull with dental cement (Tetric Evoflow). Animals were followed daily after surgery.

#### Injections in the spinal cord

Animals were anesthetized as described above and spinal injections were performed as previously done^44,74^. A two cm skin incision was performed dorsally on anesthetized animals and exposed spinal column was fixed with two holders on the left and right sides to a stereotaxic frame to minimize movements. Vertebral spinous processes were used as landmarks to target specific segments^75^. A small incision of the ligamentum Flavum allowed access to the spinal cord. A pulled glass pipette connected to a motorized syringe pump injector (Legato 130, KD Scientific, customized by Phymep, France) was positioned into the ventromedial area of the L2 (second lumbar segment, between the 11^th^ and 12^th^ vertebral bodies) using the following coordinates: 350 μm laterally from the dorsal artery and 800 μm depth from the dorsal surface. This lateral positioning ensures that the injection pipette does not pass through the lateral funiculus where ascending and descending axons travel. The volume injected was 600-750 nL/side to allow for transfection of neurons across multiple thoraco-lumbar segments (see Fig. 4a, b). For Cholera Toxin B (CTB) experiments (Fig. S8), we injected 600-750 nL of CTB-AF647 conjugate (ThermoFisher Scientific, ref. # C-34778) diluted at 0.5% in sterile water on each side of the spinal cord. After each injection, the pipette was held in place for 5 to 10 min before being slowly retracted. The skin was sutured, and animals were followed daily after the surgery. All animals recovered without motor impairments.

#### Diaphragm EMG recordings

The protocol is inspired by previous work^4^. A 12 cm pair of electrodes was prepared from Teflon-coated insulated steel wires with an outside diameter of 0.14 mm (A-M systems, ref. # 793200). Two wires were lightly twisted together, and a knot was placed 5 cm from one end. At 1 cm from the knot, the Teflon insulation was stripped over 1 mm from each wire so that the two bare regions were separated by about 2 mm. The ends of the two wires were soldered to a miniature dissecting pin. The free ends of the electrodes, and a 5 cm ground wire were soldered to a micro connector (Antelec). Nail polish was used to insulate the wires at the connector.

For diaphragm implantation, animals were anesthetized with isoflurane throughout the surgery (4% at 1 L/min for induction; 2-3% at 0.2 L/min for maintenance), placed in a stereotaxic frame and hydrated by a subcutaneous injection of saline solution (0.9%) as previously reported^4^. Their temperature was maintained at 36 °C with a feedback-controlled heating pad. This step was crucial to ensure post-surgery survival. The skull was exposed and processed to secure the micro connector using dental cement (Tetric Evofow). The ground wire was inserted under the neck’s skin and the twisted electrodes were tunneled towards the right part of the animal guided by a 10 cm silicon tube of 2 mm inner diameter. The animal was then placed in supine position, the peritoneum was opened horizontally under the sternum, extending laterally to the ribs, and the silicon tube containing the electrodes was pulled through the opening. The sternum was clamped and lifted upwards to expose the diaphragm. A piece of stretched sterile parafilm was placed on the upper part of the liver to avoid friction during movement of the animal and to prevent conjunctive tissue formation at the recording sites. The miniature dissecting pin was pushed through the right floating ribs. The pin was then inserted through the sternum, leaving the bare parts of the wires in superficial contact with the diaphragm. The electrodes’ position was secured on both sides of floating ribs and sternum using dental cement. The pin was removed by cutting above the secured wires. The peritoneum and abdominal openings were sutured and a head bar was placed on the cemented skull to facilitate animal’s handling when connecting and disconnecting EMG cables during behavioral sessions. Buprenorphine (0.025 mg/kg) was administered subcutaneously for analgesia right after surgery and animals were observed daily following the surgery and treated with Buprenorphine (0.025 mg/kg per day) if needed.

### Histology

Adult mice were anesthetized with Euthasol Vet (140 mg/kg) and perfused with 4% paraformaldehyde (PFA) in 1× Phosphate Buffered Saline (PBS). Brains and spinal cords were dissected out and fixed overnight in 4% PFA at 4 °C. After fixation, tissues were rinsed in 1× PBS. Brains and spinal cords were cryoprotected overnight at 4 °C, respectively in 16% and 20% sucrose in PBS. Tissues were rapidly cryo-embedded in OCT mounting medium and sectioned at 30 µm using a cryostat. Sections were blocked in a solution of 1× Tris Buffered Saline (TBS), 5% normal donkey serum and 1% Triton X-100. Primary antibodies, carried out 48 h at 4 °C, were: goat anti-ChAt (1:500, ref. # AB144P, Merck Millipore), chicken anti-GFP (1:500, ref. # 1020, Aves Labs), rabbit anti-RFP (1:500, ref. # 600-401-379, Rockland), rabbit anti-SST (1:500, ref. # T-4103, BMA Biomedicals), and sheep anti-TH (1:500, ref. # AB1542, Merck Millipore). Primary antibodies were detected after 2 h of incubation at room temperature with appropriate affinity-purified secondary antibodies obtained from Jackson ImmunoResearch and used at a final dilution of 1:500: donkey anti-chicken AlexaFluor 488 (ref. # 703-545-155), donkey anti-rabbit Cy3 (ref. # 711-165-152) or Cy5 (ref. # 711-175-152), donkey anti-Goat AlexaFluor 647 (ref. # 705-605-147) and donkey anti-sheep Cy 3 (ref. # 713-166-147). Sections were counterstained with a fluorescent Nissl stain (NeuroTrace 435/445 blue, ref. # N21479 or NeuroTrace 640/660 deep-red, ref. # N21483, 1:1000, Thermo Fisher Scientific) and mounted in Prolong Diamond Antifade Montant (P36970, Thermo Fisher Scientific). Sections were acquired with a Leica TCS SP8 confocal microscope running the LAS X v3.5 software (NeuroPICT imaging platform of the NeuroPSI Institute) and using ×10 and ×25 objectives, or on Zeiss AxioImager running the ZEN 3.4 software using a ×10 objective.

The preBötC was defined as located ventrally to the cholinergic ChAT^+^ neurons of the nucleus ambiguus (na) where somatostatin^+^ (SST) neurons are detected^39^ (Figs. 1, 4, 7; from 7.0 to 7.4 mm caudal to bregma). The pF respiratory region was defined as immediately ventral, ventro-median and ventro-lateral to facial motor neurons^76^ (7N; Figs. 1, 4, 6, and 7; from 6.7 to 5.7 mm caudal to bregma).

### Behavioral experiments

#### Optogenetic activations

Behavioral experiments started 3 to 5 weeks after the viral injection. Implanted animals were connected to a laser source (473 nm DPSS system, LaserGlow Technologies, Toronto, Canada) through a mating sleeve (Thorlabs). The laser was triggered by the output of a National Instruments interface (NI-USB 6211) and the timings of light activations were delivered using the NI MAX v19.6 software, as we did previously^44^. For CnF, RTN^*Phox2b/Atoh1*^, PAG or IC long photostimulation, light was delivered in trains of pulses of 20 ms (5 to 20 Hz) and of 15 ms (30 and 40 Hz) for a duration of 1 s. Each stimulation frequency was repeated three times with several minutes of rest between trials. We used the minimal laser power sufficient to evoke a response, which was measured to be between 5-12 mW at the fiber tip using a power meter (PM100USB with S120C silicon power head, Thorlabs) to restrict photoactivations unilaterally^47^, prevent heat, and exclude an unintentional silencing by over-activation. For randomized short light-pulses, 50 ms light stimulations (50-70 pulses/experiment) were applied randomly in the respiratory cycles.

#### Plethysmography recordings

To analyze the effect of short photoactivations of the CnF and CnF fibers in the preBötC (Fig. 2), RTN^*Phox2b/Atoh1*^ (Fig. 7), and PAG and IC (Fig. S5) on burst timing, ChR2-injected animals were placed inside a hermetic whole-body plethysmography (WBP) chamber^60^, customized to allow the passage of the optical patch-cord, four weeks after viral injection. The plethysmography signal was recorded over a period of 10 min using a National Instruments Acquisition card (USB-6211) and the LabScribe NI v3.0 software (iWorxs).

#### Locomotion in a linear runway

Four to five weeks following the injection of the ChR2-expressing virus in the CnF, animals were implanted with a diaphragm EMG as explained previously (Fig. 3). One week following EMG implantation, animals were placed in a linear corridor (80 × 10 cm), and familiarized for 1 h/day for 3 days prior to experiments. Implanted animals were filmed from the side at 200 fps and 0.5 ms exposure time using a CMOS camera (Jai GO-2400-USB) and images were streamed to a hard disk using the 2^nd^ LOOK v2.0 software (IO Industries). The start of the EMG recordings was hardware-triggered by the start of the video-recordings using the frame exposure readout of the video camera, so that the two recordings are synchronized. When animals were immobile at one end of the corridor and their respiration was stable, we delivered CnF optogenetic activations with frequencies ranging from 5 to 40 Hz. For each frequency, the stimulation was repeated three times with several minutes of rest between trials.

#### Chemogenetic silencing and treadmill experiments

Three weeks following the injection of the hM4Di virus in the RTN^*Phox2b/Atoh1*^, animals were implanted for diaphragm EMG recordings as explained above (Fig. 6). Non-injected C57BL/6 J wild-type mice were also implanted as controls to test for CNO side effects (Fig. S11). One week following EMG implantation, animals were familiarized on a stationary custom-made motorized treadmill with adjustable speed range (Scop Pro, France, belt dimensions: 6 cm × 30 cm) for 30 min/day for 3 days prior to the experiments. In addition, implanted animals were exercised during this time for a total of 5 min at 40 cm/s each day. Mice could rest for 5 min after running before being placed back in their cage. This step was crucial to obtain stable running animals during experimental sessions. Following this short training, implanted mice were connected with custom light-weight cables to an AC amplifier (BMA-400, CWE Inc.) and neurograms were filtered (high-pass: 100 Hz, low-pass: 10 kHz), collected at 10 kHz using a National Instruments acquisition card (USB-6211) and live-integrated using the LabScribe NI v3.0 software (iWorxs). Animals were first placed on the stationary treadmill to monitor basal respiration. Animals were then challenged to trot at 40 cm/s for 1.5 min before being administered intraperitoneally with CNO (Enzo Life science, ref. #: BML-NS105-0005) at 10 mg/kg^61^ or saline (0.9%). Animals were placed again on the treadmill with the same paradigm 2-3 h and 5 h after CNO or saline administration to measure respiration in resting and running conditions. During experiments, animals were filmed from the side in the same way as above to monitor the stability of running episodes.

#### Ex vivo brainstem-spinal cord experiments

Pups aged 1-2 days were used in all experiments. Neonates were anaesthetized with isoflurane, decerebrated and the brainstem still attached to the spinal cord was dissected and isolated in ice-cold Ringer’s solution that contained (in mM): 111 NaCl, 3 KCl, 25 NaHCO_3_, 1.25 MgSO_4_, 1.1 KH_2_PO_4_, 2.5 CaCl_2_ and 11 D-Glucose, and oxygenated in 95% O_2_, 5% CO_2_ to obtain a pH of 7.4. Isolated brainstem-spinal cords were transferred into a recording chamber and pinned to a Sylgard 184 resin. Preparations were partitioned in two compartments at the level of lower thoracic segments (T11) using a Vaseline wall, to restrict bath application of locomotor drugs on the lumbar spinal cord (Fig. 5). The lumbar compartment was continuously perfused with the above Ringer’s solution while the rostral compartment containing the brainstem was superfused with a Ringer’s solution that contained (in mM): 111 NaCl, 8 KCl, 25 NaHCO_3_, 3.7 MgSO_4_, 1.1 KH_2_PO_4_, 1.25 CaCl_2_ and 30 D-Glucose. All recordings were done at room temperature (25 °C) after allowing 30 min recovery period after the dissection. Respiratory- and locomotor-like activities were recorded respectively on the 4^th^ cervical (C4) and the 2^nd^ lumbar (L2) ventral roots using extracellular suction glass pipettes (120F-10, Harvard Apparatus). Drug-evoked locomotor-like activities were induced by bath-applying 10 to 14 µM of N-methyl-D-aspartate (NMDA, Tocris) and serotonin (5-HT, Sigma-Aldrich, Fig. 5), or using blue light on the lumbar spinal cord of ChR2-expressing pups (*Vglut2*^*Cre*^*; ChR2* ^*floxed*^, Fig. S9d–f). Signals were collected and band-passed filtered at 100 Hz to 1 kHz with an AC amplifier (Model 1700, A-M Systems) and live-integrated (Neurolog NL703, Digitimer) with a time constant of 100 ms (C4) or 200 ms (L2). Signals were sampled using Clampex 11 (Molecular Devices) at 5 kHz. To control for locomotor drugs leakage, some preparations were transected at the level of the cervical spinal cord (Fig. S9a–c). For brainstem transection experiments, the rostral part of the brainstem containing the pF respiratory region was physically removed (Fig. 5d–f). *Egr2* ^*Cre*^*;Phox2b* ^*27AlaCKI*^ pups were used to genetically eliminate RTN^*Phox2b/Atoh1*^ neurons^59,60^ (Fig. 5g–i).

### Quantifications and statistical analysis

#### Registration of CnF injection sites

To evaluate the extent of viral expression in the CnF and neighboring areas (anterograde tracing, Fig. 1; photostimulations, Figs. 2 and 3), we registered the position of all infected cells using ImageJ Cell Counter plugin from one coronal plane at sites of injection. The position of reporter-positive cells from each coronal section as well as optic fiber placement are shown in Fig. S1.

#### Axonal projection and cell quantifications

To quantify axonal projections from CnF (Fig. 1g), lumbar (Fig. 4e), and RTN^*Phox2b/Atoh1*^ (Fig. 7d) neurons to brainstem respiratory areas, we manually defined i) the pF region as immediately ventral, ventro-median, ventro-lateral and lateral to facial motor neurons^76^ (bregma − 6.7 to −5.7 mm), and ii) the preBötC as a 400 × 400 μm square residing ventrally to the ChAT^+^ nucleus ambiguus, where somatostatin^+^ cells are detected^39^ (bregma −7.0 to −7.4 mm). We used three rostro-caudal coronal sections from preBötC and/or pF and measured the number of fluorescent pixels per μm^2^ bilaterally (Figs. 1 and 7) or unilaterally (Fig. 4) using the ImageJ Measure plugin. All values were averaged across animals (3 sections/animal) and a grand mean ± SD across n animals was calculated per hemi-section.

To evaluate the rostro-caudal organization of projections and transfected cells within the pF, we delineated pF subregions as follows: bregma −5.7 (rostral), −6.1 (intermediate), and −6.7 (caudal, see Fig. 4f). Each pF subregion was further divided into lateral vs medial portions. We used one coronal section per subregion per animal to quantify i) projections from the lumbar spinal cord (Fig. 4f) using ImageJ Measure plugin, and ii) the number of infected pF (Fig. 4i) and RTN^*Phox2b/Atoh1*^ (Figs. 6b and 7b) neurons using ImageJ Cell Counter. All values were averaged across animals and a grand mean ± SD across n animals was calculated per hemi-section.

To assess the rostro-caudal distribution of transfected cells in the spinal cord (Fig. 4b), we used one coronal section from seven segments of the spinal cord: the 12th (T12) and 13^th^ (T13) thoracic, and the 1st to 5th lumbar (L1 to L5) segments. Cells were counted using ImageJ Cell Counter. All values were averaged across animals (1 section/animal) and a grand mean ± SD across n animals was calculated per hemi-section.

#### Phase-shift analysis

The durations of the respiratory cycle containing the light stimulus (perturbed cycle, θ) and the previous respiratory cycle (control cycle, ɸ, Figs. 2 and 7) were measured. One respiratory cycle was defined from the onset of inspiration to the subsequent inspiratory onset. The phase of light-stimulation ɸ_S_ was defined from the onset of the perturbed cycle to the onset of the light pulse. The perturbed cycle θ was defined as from the onset of the inspiration that precedes the light stimulation to the onset of the subsequent inspiration. The perturbed phase (phase-shift) was calculated as the ratio of the perturbed cycle divided by the control cycle (θ/ɸ). The light phase was defined as the ratio of the stimulated cycle divided by the control cycle (ɸ_S_/ɸ). The perturbed phase was then plotted against the light phase for all events from all animals. A phase shift <1 (perturbed cycle duration lower than the control one) indicates a shortening of the respiratory cycle, a phase shift >1 (perturbed cycle duration higher than the control) indicates a lengthening, and a phase shift equal to 1 (perturbed cycle duration equal to the control) indicates no effect. The number of events (N) and animals (n) are given in the corresponding figures for all tested condition. In addition, the average perturbed phase was plotted against the average light phase in 0.1 ms bins as mean ± SD. Inspiratory time (I) was measured, averaged for each animal and a grand average was calculated and annotated in the corresponding figures for all tested condition. Expiratory time (E) was calculated from respiratory cycle and inspiratory (I) times.

#### Locomotor parameters analysis

To track the mouse displacement and measure its speed, we used DeepLabCut (version 2.1.5.2, ref. ^48^) and manually labeled the positions of the head from 50 frames of each video. We then used 95% of the labeled frames to train the network using a ResNet-50-based neural network with default parameters for 3 training iterations. This network was then used to analyze videos from similar experimental settings. For treadmill experiments (Fig. 6), the head x coordinate was used as a control for running stability on the treadmill. For CnF stimulations on the corridor (Fig. 3), the head x coordinate was used to calculate the animal’s speed s_x_ using the gradient over time.

$$\vec{{s}_{x}}=\frac{\partial \vec{x}}{\partial t},\, \vec{x}\,{{{{{\rm{being}}}}}}\; {{{{{\rm{the}}}}}}\; {{{{{\rm{displacement}}}}}}\; {{{{{\rm{of}}}}}}\; {{{{{\rm{the}}}}}}\; {{{{{\rm{head}}}}}}\; {{{{{\rm{along}}}}}}\; {{{{{\rm{the}}}}}}\; {{{{{\rm{x}}}}}}\; {{{{{\rm{axis}}}}}}$$

Head X coordinate (treadmill) and calculated velocity (corridor) were then exported to Clampfit (Molecular Devices) and interpolated to 10 kHz to match the acquisition rate of diaphragmatic EMG recordings, as we did previously^4^. Both sets of signals (head X and diaphragm, or speed and diaphragm) were merged in single files, before being processed offline in Clampfit. The animal’s instantaneous speed is illustrated on Fig. 3. The mean speed, defined from movement onset to the end of the photostimulation, was then calculated using the statistic function in Clampfit for each CnF stimulation (5 to 40 Hz). All values were averaged across trials for each animal (3 trials/animal), and a grand mean ± SD across n animals was calculated per stimulation frequency (Fig. S6b).

Locomotor onset delay (Fig. S6c) was defined as the latency between the onset of the CnF stimulation and the onset of movement for each CnF stimulations. All values were averaged across trials (3 trials/animal) and a grand mean ± SD across n animals was calculated per stimulation frequency.

For gait analysis during CnF photostimulations (Fig. S6d), we manually annotated the paw of a reference hindlimb (ipsilateral) and registered the timings of footfalls (when the paw first touches down). Each reference locomotor cycle was then defined as the duration from one footfall (ipsi_FF_n_) to the next (ipsi_FF_n+1_). The time of occurrence of the contralateral hindlimb footfall within the reference locomotor cycle was annotated manually (contra_FF) and the synchronicity rate was then computed as follows:

$${synchronicity}\,{rate}=\frac{{t}_{{contra}\_{{{{{\rm{FF}}}}}}}-{t}_{{{{{{\rm{ipsi}}}}}}\_{{{{{\rm{FFn}}}}}}}}{{t}_{{{{{{\rm{ipsi}}}}}}\_{{{{{\rm{FFn}}}}}}+1}-{t}_{{{{{{\rm{ipsi}}}}}}\_{{{{{{\rm{FFn}}}}}}}}}$$

Ipsilateral and contralateral hindlimb steps were categorized as synchronized (synchronicity rate ∈[0, 0.25[∪[0.75, 1]) or alternated (synchronicity rate ∈[0.25, 0.75[). Synchronicity rates were averaged across animals (3 trials/animal) and a grand mean ± SD across n animals was calculated per stimulation frequency.

#### Locomotor/respiratory coordination

The temporal coordination of breaths to strides (Fig. S7) was represented with circular statistics, similarly to what we performed recently^4^ and imprinting from numerous studies having investigated the cycle-to-cycle correlations of motor activities^54,55,77^. The phase of each individual inspiratory burst within the locomotor cycle (ΦInsp, around 15 bursts) is represented as the position, from 0 to 1, of diamond marks on the outer circle (see Fig. S7d). For each animal, we also computed the mean phase of consecutive inspiratory bursts and represented it as a colored circle (the mean phases of different animals are in different colors). The distance R of the mean phase to the center of the circle indicates the concentration of individual phase values around the mean, as established by^55^. If inspiratory and locomotor movements are temporally correlated, then individual phase values will be concentrated around a preferred phase value (for instance 0 or 1, at the top of the circle, if the two motor activities were in phase). The mean value would then be positioned at a significant distance from the center. Conversely, if inspiratory and locomotor movements are not coupled, individual phases will be evenly distributed across the circle. Consequently, the mean phase value will be at a short distance from the diagram center, illustrating the dispersion of values around the mean. The inner circles of the circular diagrams depict the threshold for mean phase values to be considered significantly oriented (R < 0.3) as commonly done^4,54,55,77^. Circular plots were obtained using a custom macro in Excel.

#### In vivo respiratory changes analysis

To analyze breathing changes resulting from CnF (EMG recordings; Fig. 3), RTN^*Phox2b/Atoh1*^, PAG and IC photostimulation (WBP recordings; Figs. 7 and S5), instantaneous inspiratory frequencies and amplitude were detected over a 1 s window, using the threshold search in Clampfit before, during and directly after a 1 s light stimulation for all frequencies (5 to 40 Hz). For CnF stimulations that triggered locomotor episodes (15 to 40 Hz), the recovery period was measured as soon as the animal returned to immobility. Breaths detected from the onset of the light stimulus to the onset of movement were categorized as “pre-loco” phase (Fig. 3d, e). Breaths detected from movement onset to the offset of the light stimulus were categorized as “loco” phase (Fig. 3d, f). All values were averaged across animals (3 trials/animal) and a grand mean ± SD across n animals was calculated per stimulation frequency.

For treadmill running (Fig. 6), the mean diaphragm frequency was analyzed prior to exercise (resting condition) and from stable trotting moments, i.e., when the animal’s speed was in phase with the treadmill, inferred by the absence of changes in head’s X coordinates (running condition) ^4^. For each condition, i.e., before (CTL), during (CNO/saline) and after (REC) administration of either CNO or saline, we measured instantaneous respiratory frequency and amplitude using the threshold search in Clampfit. Inspiratory (Ti) and expiratory (Te) times were quantified manually before (CTL), during (CNO/saline) and after (REC) administration of either CNO or saline. These measurements were done using 2 to 3 windows of 6 s each taken during resting conditions and at any stable moment of the 1.5 min run (excluding the first 20 s to avoid possible stress-induced changes when the treadmill is just engaged). Measurements were averaged to give the mean value for each animal. Averaged mean values were expressed as mean ± SD across n animals.

#### Ex vivo respiratory-like activities analysis

Instantaneous respiratory-like frequencies were analyzed offline using the threshold search in Clampfit (Molecular Devices) before, during and after bath application of NMDA and 5-HT (Fig. 5). Respiratory frequency changes during drug and washout conditions were normalized and expressed as a percent of control values. A grand mean ± SD across n animals was calculated.

#### Statistical analysis

All data are expressed as mean ± SD and graphs were produced in MATLAB (R2021a) or Microsoft Excel. Two-tailed statistical tests were performed using GraphPad Prism 7 and are spelled out in figure legends, as well as the number of trials (N) and animals (n) used for each experiment. Changes were considered as not significant (ns) when p > 0.05 and as significant when p < 0.05. Exact p values are reported between conditions (*) and with control condition (§; Fig. 7) in corresponding figures.

### Reporting summary

Further information on research design is available in the Nature Portfolio Reporting Summary linked to this article.

## Supplementary information

Supplementary Information

Reporting Summary

## Data Availability

The data that support the findings can be found in the source data provided with the paper. Original microscopy data have been deposited to Mendeley Data (10.17632/wpbyxgfr96.1). For other raw data, example files can be obtained upon request. Source data are provided with this paper.

## Source data

Source Data
