# Supplementary material for: Upregulation of breathing rate during running exercise by central locomotor circuits in mice
Source: Nat Commun. 2023 May 22;14:2939. doi: 10.1038/s41467-023-38583-6 (PMC10203288; doi:10.1038/s41467-023-38583-6)
Supplement: Supplementary file 1 — Supplementary Information [file 41467_2023_38583_MOESM1_ESM.pdf]

Upregulation of breathing rate during running exercise by central locomotor circuits in mice

Coralie Hérent[1,3], Séverine Diem[1,4], Giovanni Usseglio[1], Gilles Fortin[2] and Julien Bouvier[1*]

[1]Université Paris-Saclay, CNRS, Institut des Neurosciences Paris-Saclay, 91400, Saclay, France.

[2]Institut de Biologie de l'École Normale Supérieure (IBENS), École Normale Supérieure, CNRS, INSERM, PSL Research University, 75005, Paris, France.

[3]Present address: Champalimaud Research, Champalimaud Foundation, 1400-038, Lisbon, Portugal.

[4]Present address: Institute of Functional Genomics, University of Montpellier, CNRS, INSERM, 34094 Montpellier, France.

*Correspondence: julien.bouvier@cnrs.fr

Supplementary Figures 1 to 12

Supplementary Figure 1

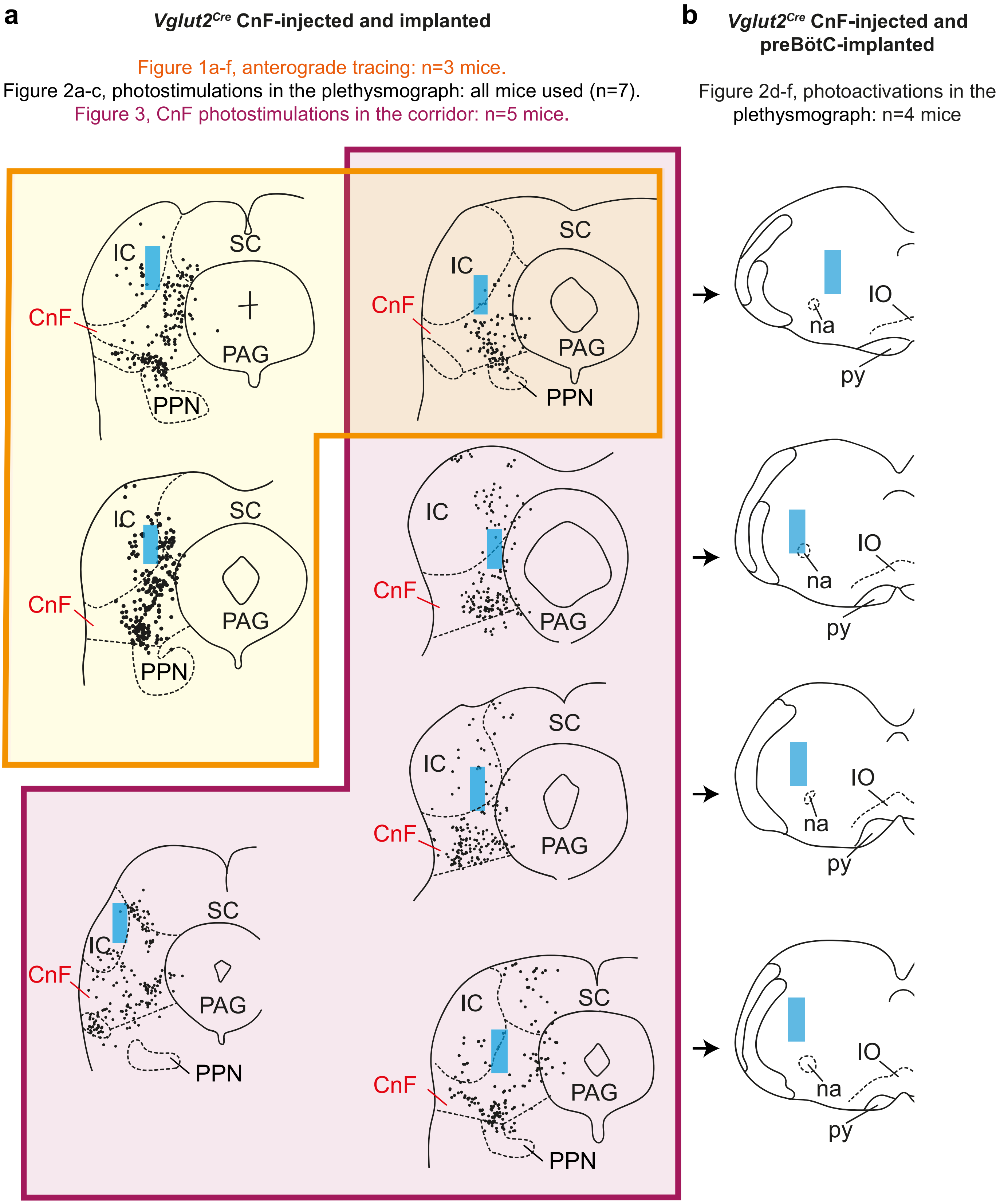

Supplementary Figure 1. Viral transfection and optic fiber implantation sites in the CnF and preBötC. Related to Figures 1, 2 and 3.
(a) Registration of transfected glutamatergic CnF cells (dots) and optic fiber placements (blue rectangles) in *Vglut2*^{Cre} adult mice used for Figures 1a-f (orange), 3 (pink), and 2a-c (all animals). One section represents one animal.
(b) In the 4 animals indicated by an arrow from (**a**), an additional optic fiber was placed in the ipsilateral preBötC to activate CnF projections in this region (experiments shown in Figure 2d-f).

Supplementary Figure 2

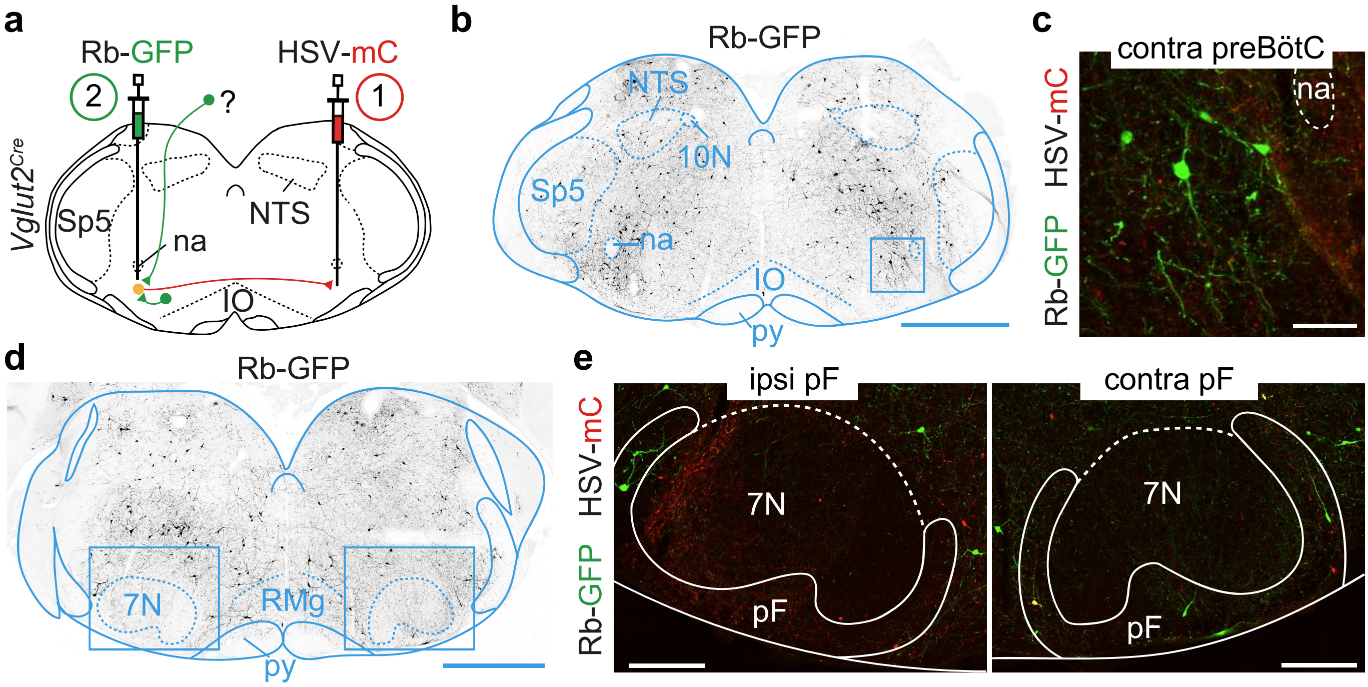

Supplementary Figure 2. Transsynaptic labelling from glutamatergic commissural preBötC neurons leads to only little labelling in the pF. Related to Figure 1.
(a) Experimental design for retrograde transsynaptic monosynaptic tracing from Glut^{+} commissural preBötC neurons in *Vglut2^{Cre}* adult mice. An HSV-LS1L-TVA-oG-mCherry (HSV-mC) is first injected in the contralateral preBötC followed by a G-deleted and EnvA pseudotyped Rb virus (Rb-GFP) in the ipsilateral preBötC.
(b) Transverse section at the preBötC level showing Rb-GFP putative presynaptic cells. Scale bar, 1 mm.
(c) Magnification over the contralateral preBötC showing Rb-GFP putative presynaptic cells. Scale bar, 100 µm.
(d) Transverse section at the level of the pF showing the scarcity of Rb-GFP presynaptic cells. Scale bar, 1 mm.
(e) Magnification over ipsilateral and contralateral pF areas. Scale bars, 250 µm.
All panels are representative of n = 3 animals.

Supplementary Figure 3

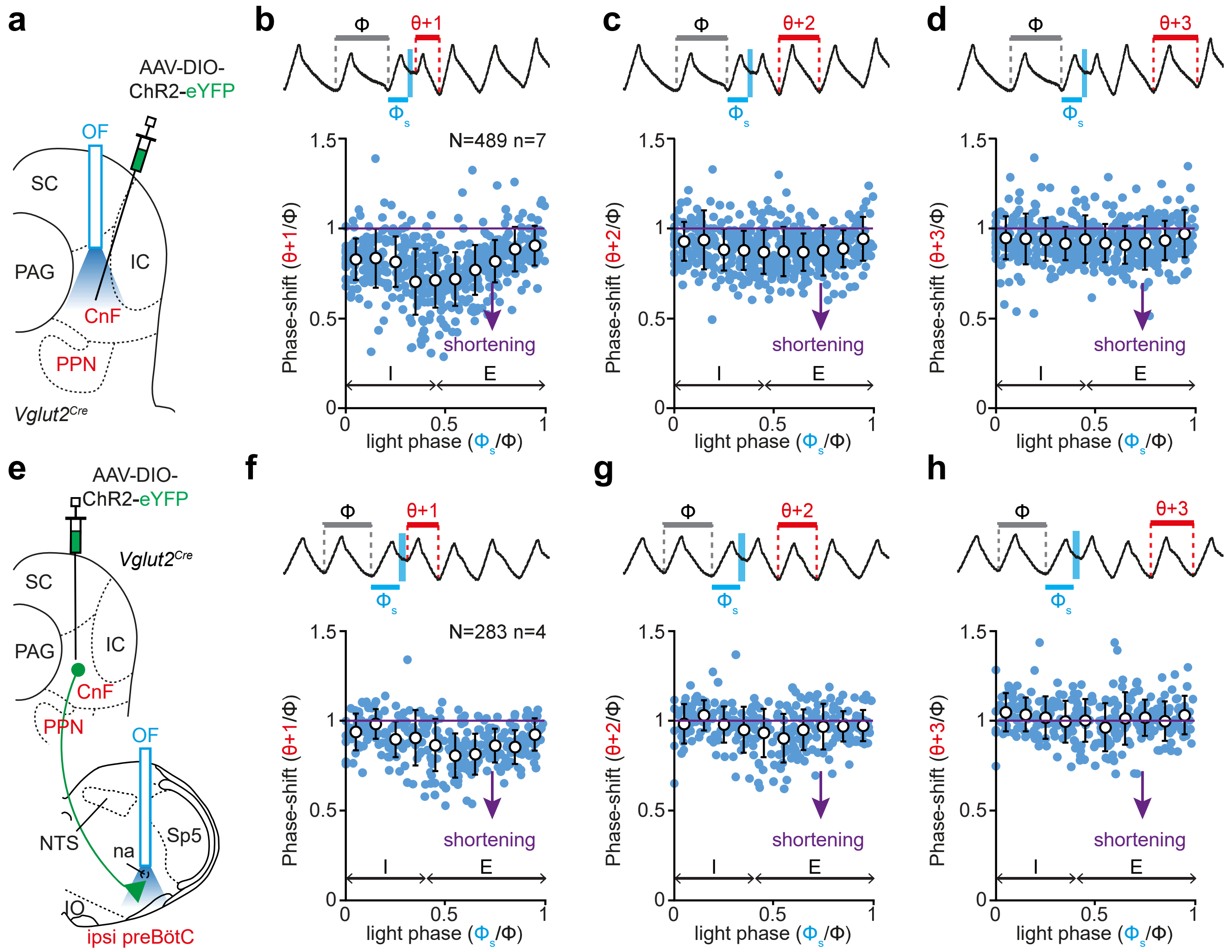

Supplementary Figure 3. Impact of photoactivating glutamatergic CnF neurons and their projection to the preBötC on respiratory cycles following the light-perturbed cycle. Related to Figure 2.
(a) Experimental strategy for photoactivating Glut+ CnF neurons on a *Vglut2Cre* adult mouse.
(b) *Top*: whole body plethysmography (WBP) recordings of respiratory cycles around a single 50 ms photoactivation of Glut+ CnF neurons (inspirations are upwards, expirations are downwards). The control cycle (Φ, black), the phase of light-simulation ($Φ_s$, blue), and the cycle following the perturbed cycle (θ+1, red) are annotated. *Bottom*: plot of the phase-shift (cycle n+1 normalized to control cycle: θ+1/Φ) as a function of light phase (light cycle normalized to control cycle: $Φ_s$/Φ). Values < 1 (purple line) indicate a shortening of the cycle n+1. Blue circles represent individual data from N random trials from n mice and white circles are averages ± SD across all trials within 0.1 ms bins. Inspiration (I) and expiration (E) mean durations are annotated. See Figure 2 for details.
(c, d) Similar representations for cycles n+2 (θ+2, **c**) and n+3 (θ+3, **d**).
(e) Experimental strategy for photoactivating Glut+ CnF projections in the preBötC on a *Vglut2Cre* adult mouse.
(f-h) Similar representations as in (**b-d**) when activating Glut+ CnF fibers in the preBötC.
For all graphs, source data are provided as a Source Data file.

Supplementary Figure 4

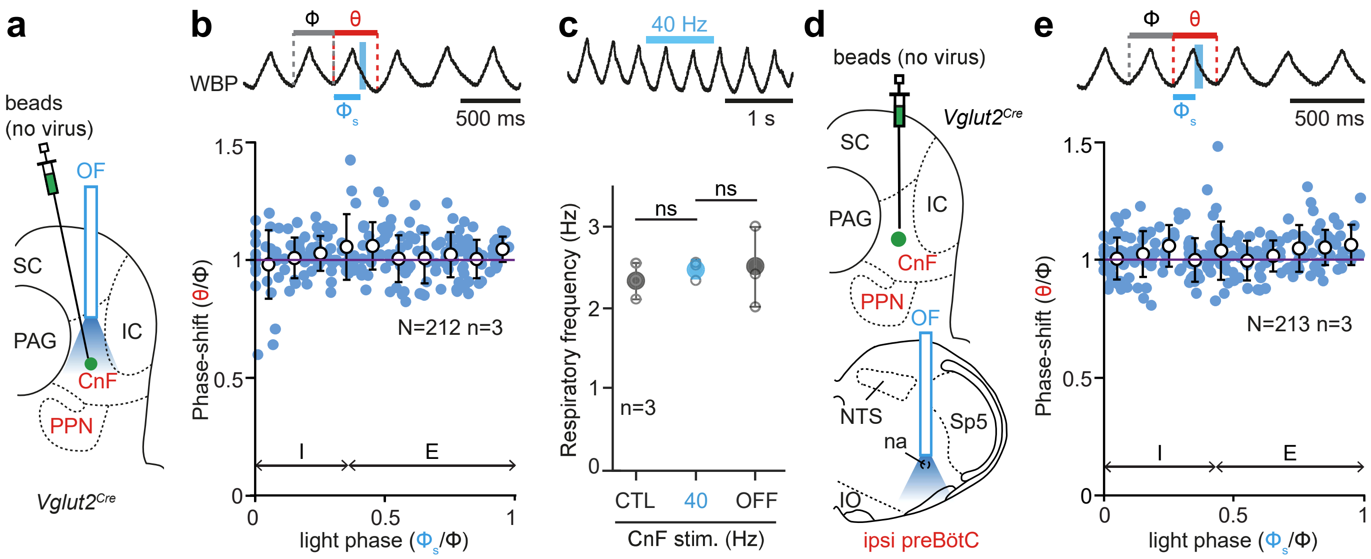

Supplementary Figure 4. Controls for optogenetic activations of glutamatergic CnF neurons. Related to Figures 2 and 3.

(a) *Vglut2cre* adult mice were injected with fluorescent beads in the CnF, but no ChR2-coding virus. An optic fiber (OF) was implanted above the injection site.

(b) *Top*: whole body plethysmography (WBP) recordings of respiratory cycles around a single 50 ms light pulse delivered on control mice during the expiratory phase of one respiratory cycle (inspirations are upwards, expirations are downwards). The control cycle (Φ, black), the phase of light-simulation ($Φ_s$, blue), and the perturbed cycle (θ, red) are annotated (see Figure 2 for details). *Bottom*: plot of the phase-shift (perturbed cycle normalized to control cycle: θ/Φ) as a function of the phase of light-stimulation ($Φ_s$/Φ). Inspiration (I) and expiration (E) mean durations are indicated. Contrary to CnF light activations (see Figure 2), the respiratory cycle is not affected here in control mice (values close to 1, purple line). Blue circles represent individual data from N random trials from n mice. White circles are averages ± SD across all trials within 0.1 ms bins.

(c) *Top*: example WBP recording during a 1 s photoactivation (40 Hz train). *Bottom*: quantification of the diaphragm frequency showing no significant effect of the photoactivation in control mice (CTL: control; OFF: after light offset). Gray open circles are the means of individual animals and colored circles are the means ± SD across n mice. ns, p > 0,9999; Wilcoxon matched-pairs tests.

(d) Similar experimental design as in (**a**) but animals were implanted with an OF above the ipsilateral preBötC.

(e) Same as in (**b**) during a single 50 ms light delivery in the preBötC area. Contrary to light activations of CnF projections in the preBötC (see Figure 2), the respiratory cycle is not affected by photoactivations on these control mice (values close to 1, purple line).

For all graphs, source data are provided as a Source Data file.

Supplementary Figure 5

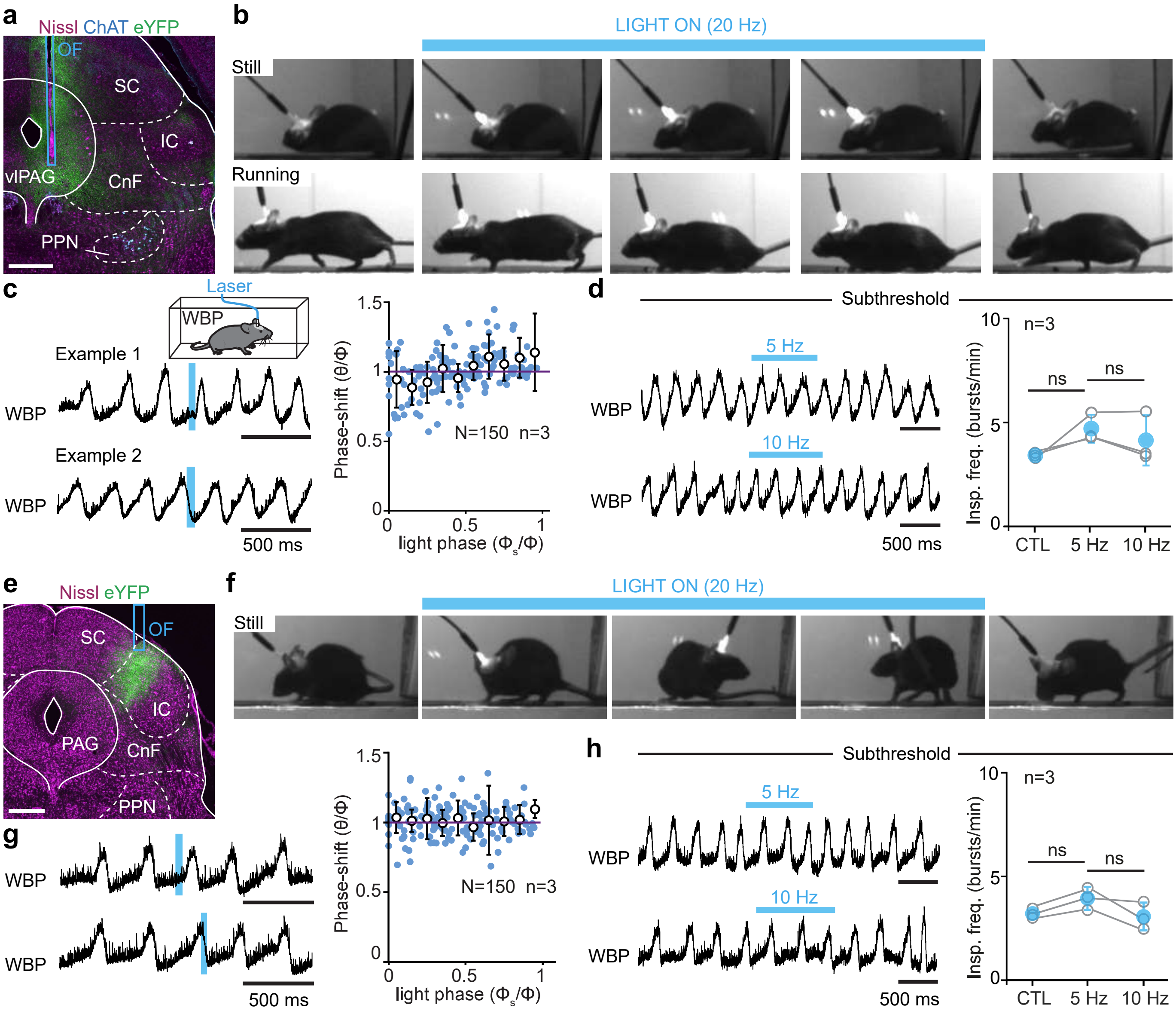

Supplementary Figure 5. Targeted photoactivations of adjacent structures to the CnF do not induce running nor modulate inspiratory rhythm mechanisms. Related to figures 2 and 3.

(a) Transverse section showing Glut$^+$ ventrolateral periaqueductal gray (vlPAG) neurons, that reside more laterally but at the same dorso-ventral depth as the CnF, unilaterally-transfected with an AAV-DIO-eYFP on a *Vglut2^{Cre}* adult mouse. Scale bar, 0.5 mm. Representative of n = 3 mice.

(b) Photostimulation of Glut$^+$ vlPAG neurons does not evoke running on resting mice and supresses running in moving mice.

(c) *Left*: WBP setup (top) and two example recordings (bottom) around a single 50 ms light stimulation of Glut$^+$ vlPAG neurons at different phases of the respiratory cycle. *Right*: plot of the phase-shift as a function of the phase of light-stimulation. See Figure 2c for details. Note that the stimulation does not significantly perturb the respiratory cycle.

(d) *Left*: two examples of WBP recordings during vlPAG photostimulation at 5 and 10 Hz showing the absence of changes in breathing frequency. *Right*: quantifications of inspiratory frequency in control (CTL), and during the vlPAG photostimulation. Gray open circles are means of 3 trials per animal and colored circles are means ± SD across n mice. ns: not significant, paired t-tests. Exact p values from left to right: p = 0,1027; p = 0,2134.

(e-h) Similar representations for targeted photostimulation of Glut$^+$ neurons of the inferior colliculus (IC), that resides more dorsally, but at the same medio-lateral location, as the CnF. Representative of n = 3 mice. The photostimulation evokes a contralateral turning but no locomotor initiation in resting mice, and does not modulate inspiratory rhythm. ns: not significant, paired t-tests. Exact p values from left to right: p = 0,1332; p = 0,3886.

For all graphs, source data are provided as a Source Data file.

Supplementary Figure 6

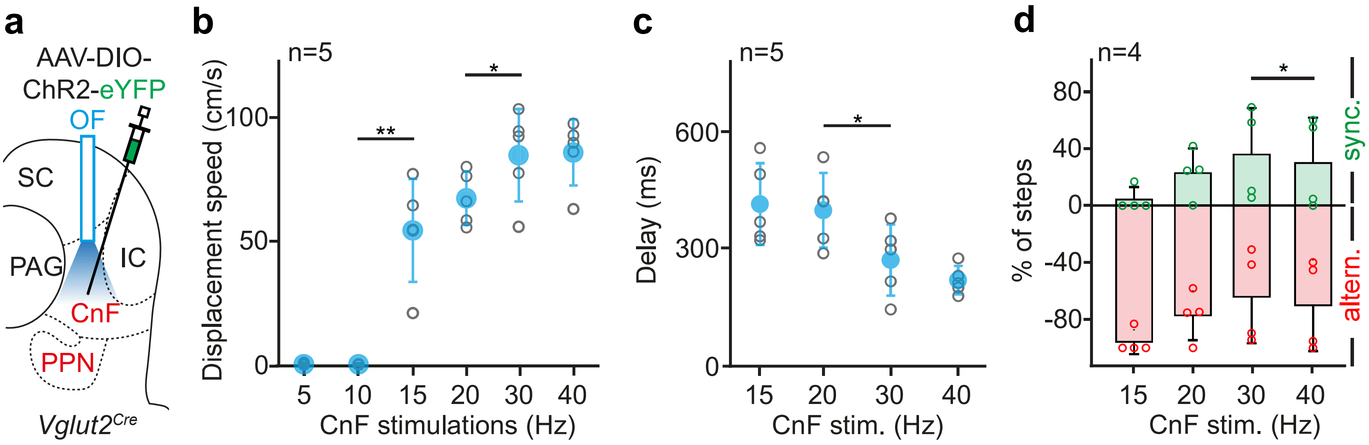

Supplementary Figure 6. Glutamatergic CnF neurons control locomotor speed, locomotor onset delay and hindlimb synchronicity rate. Related to Figure 3.

(a) Experimental strategy for photostimulating Glut⁺ CnF neurons in an adult *Vglut2*^{Cre} mouse.

(b, c) Quantification of the animal's displacement speed (**b**) and the delay to locomotor initiation (**c**) at increasing CnF photostimulations frequencies. Gray open circles are the means of individual animals and colored circles are the means ± SD across n mice. *, p < 0.05; **, p < 0.01, paired t-tests. Exact p values are, from left to right: **b**: p = 0,6904, p = 0,0043; p = 0,1896; p = 0,0285; p = 0,7253; **c**: p = 0,6452; p = 0,0309; p = 0,3044; .

(d) Two-sided bar-graphs showing the percent of hindlimb movements that show left-right alternation (negative values in red, indicative of trot) or left-right synchronicity (positive values in green, indicating of gallop) at increasing CnF photostimulation frequencies. Colored open circles are the means of individual animals and bar-graphs are the means ± SD across n mice. *, p < 0.05; wilcoxon matched-pairs tests. Exact p values are, from left to right: p = 0,25; p = 0,3545; p = 0,0128.

Source data are provided as a Source Data file.

Supplementary Figure 7

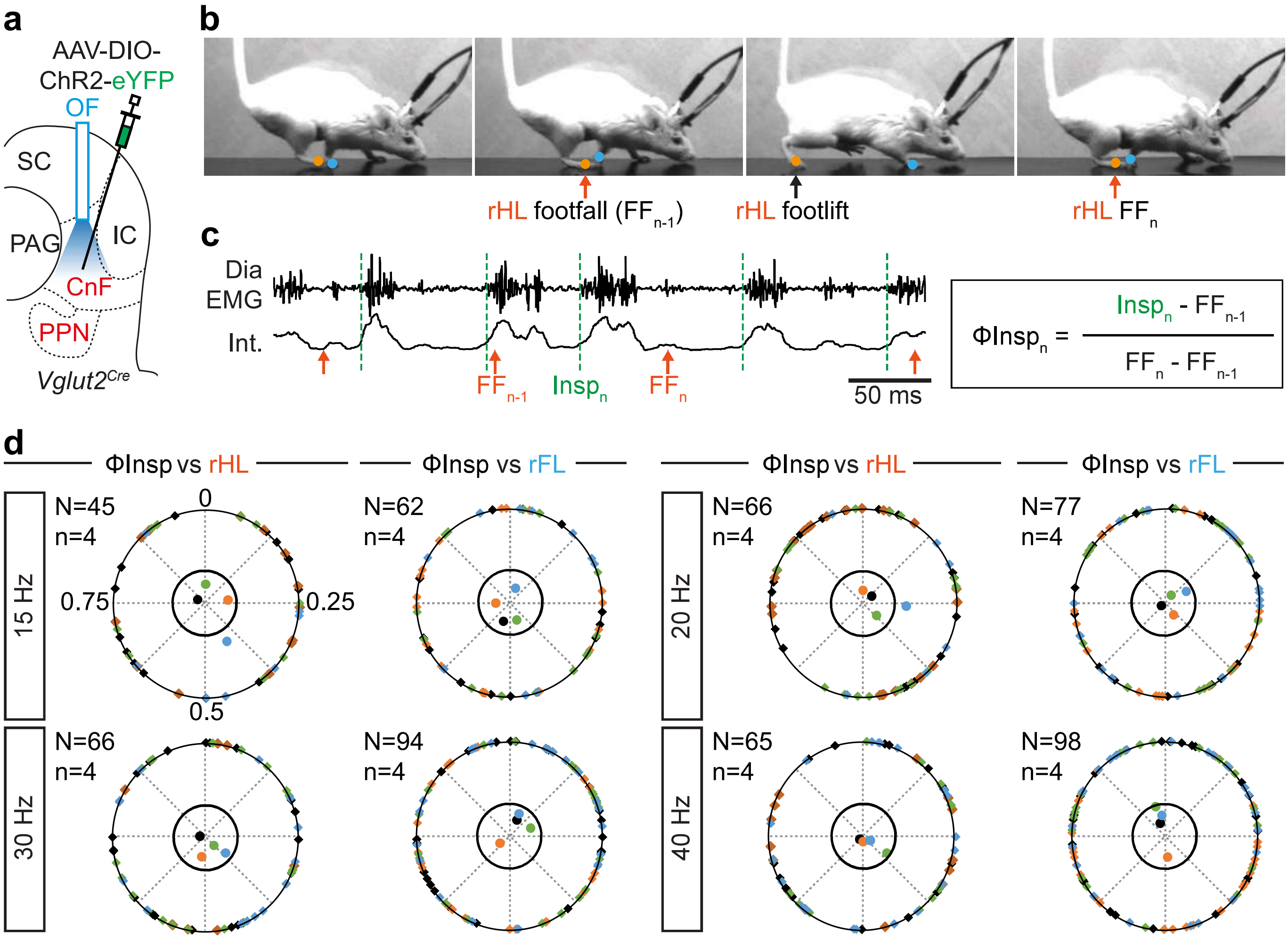

Supplementary Figure 7. Absence of phasing of respiratory and locomotor rhythms during CnF-evoked running. Related to Figures 2 and 3.

(a) Experimental strategy for photostimulating Glut^{+} CnF neurons in an adult *Vglut2^{Cre}* mouse.

(b) Photostimulation of Glut^{+} CnF neurons for 1 s at 20 Hz. The timing of footlifts and footfalls of the right hindlimb (rHL, orange) and the right forelimb (rFL, blue) were detected manually on the videos. One complete rHL locomotor cycle is shown, between two consecutive footfalls (FF_{n-1} and FF_n).

(c) Raw (EMG) and integrated (Int.) diaphragmatic electromyograms and occurrence of rHL footfalls (orange arrows) during CnF-evoked running. Dotted green lines indicate the onsets of inspirations, which can occur at any moment of the locomotor cycle. Occurrences of inspiratory bursts ($Insp_n$) within the locomotor cycle are then calculated and expressed as a phase value ($\Phi Insp_n$) from 0 (concomitant with FF_{n-1}) to 1 (concomittant with FF_n).

(d) Circular plots diagrams showing the phase-relationship between individual inspiratory bursts and the indicated reference limb for one representative animal in response to CnF photoactivations at 15, 20, 30, and 40 Hz. Diamonds on the outer circle indicate the phase of N individual inspirations pooled from n mice; values from each animal are given in the same color. Dots inside the circle indicate the mean orientation vector for each animal. The positioning of these mean values within the inner circle illustrates the absence of a significantly oriented phase preference ($R < 0.3$, R being the concentration of phase values around the mean as defined in Kjaerulff and Kiehn, 1996). Source data are provided as a Source Data file.

Supplementary Figure 8

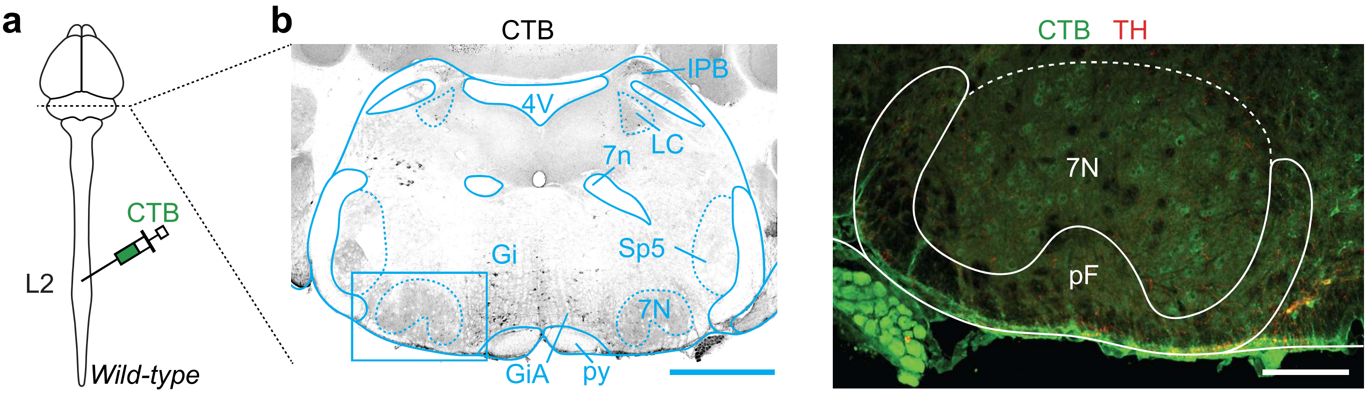

Supplementary Figure 8. Absence of spinally-projecting neurons in the pF. Related to Figure 4.
(a) Experimental strategy for labelling spinally-projecting neurons using a bilateral injection of the retrograde tracer Cholera Toxin B (CTB) at the second lumbar segment (L2) in a wild-type adult mouse.
(b) *Left*: transverse section at the level of the pF, showing CTB$^+$ spinally-projecting neurons in black. Scale bar, 1 mm. *Right*: magnification of the boxed area showing the absence of spinally-projecting neurons in the pF. CTB$^+$ cells are located more medially and include catecholaminergic cell-types (expressing tyrosine hydroxylase, TH). Scale bar, 200 µm. Representative of n = 3 animals.
lPB: lateral para-brachial nucleus. LC: locus coeruleus. See Figure 1 for other abbreviations.

Supplementary Figure 9

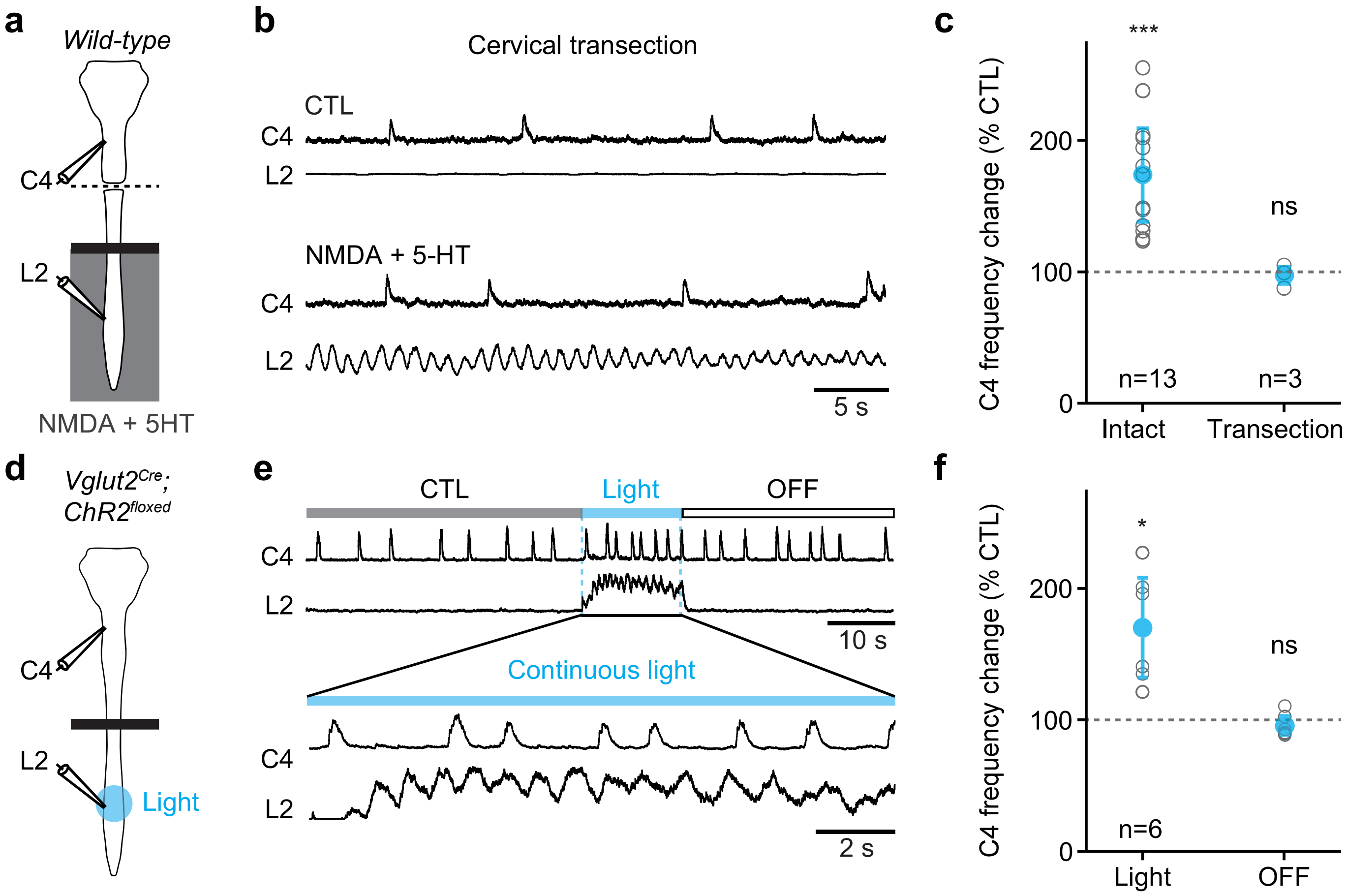

Supplementary Figure 9. Controls for ex vivo experiments and photoactivations of the glutamatergic lumbar spinal neurons. Related to Figure 5.

(a) Experimental strategy for recording respiratory (C4) and locomotor-like (L2) activities on ex vivo isolated brainstem-spinal cord preparations from neonatal wild-type mice. Preparation are separated in two compartments using a Vaseline barrier (black bar). The lumbar compartment is superfused with control or locomotor drugs enriched (NDMA and 5-HT) artificial cerebrospinal fluid (aCSF), while the brainstem compartment remains in control aCSF. Contrary to experiments in Figure 5, preparations underwent a complete transection at the cervical level.

(b) Recordings of respiratory and locomotor-like activities of one representative animal before (CTL) and during perfusion of NMDA and 5-HT in the lumbar compartment (only integrated traces are shown). The absence of change in C4 frequency following the transection rules out any leakage of NMDA and 5-HT from the lumbar to the brainstem compartment.

(c) Quantification of the respiratory frequency change during drug-induced locomotor-like activity as a percent change to CTL, in both intact and sectioned preparations. Grey open circles are the means of individual preparations, and blue circles are the means ± SD across n preparations. ***, p = 0,0002; ns, p = 0,75; wilcoxon matched-pairs tests. Source data are provided as a Source Data file.

(d) Experimental strategy for photostimulating lumbar Glut^{+} neurons in *Vglut2^{Cre};ChR2^{floxed}* neonatal ex vivo preparations. The blue light is delivered focally on the L2 segment, bilaterally.

(e) Recordings of respiratory and locomotor-like activities of one representative preparation before (CTL), during (Light) and after (OFF) a continuous 15 s photostimulation which triggers a substantial increase in respiratory frequency.

(f) Quantification of the respiratory frequency change during photo-evoked locomotor-like activity as a percent change to the CTL. Grey open circles are the means of individual preparations, and blue circles are the means ± SD across n preparations. *, p = 0,0313; ns, p = 0,3125; wilcoxon matched-pairs tests. Source data are provided as a Source Data file.

Supplementary Figure 10

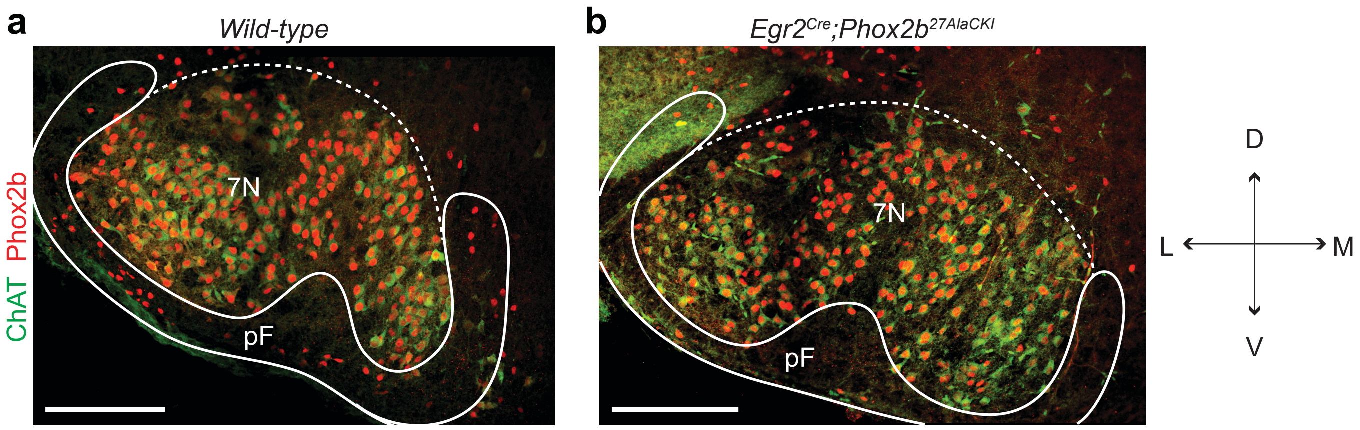

Supplementary Figure 10. Loss of RTN neurons in *Egr2^{Cre};Phox2b^{27AlaCKI}* mutant neonates. Related to Figure 5.

(a) Magnification of the pF area on a transverse brainstem section of a wild-type neonatal mouse. RTN neurons are detected as *Phox2b*-expressing interneurons located in the pF area lying ventrally, ventro-medially, ventro-laterally and laterally to the facial motoneurons (7N) which also co-express the Choline Acetyl Transferase (ChAT).

(b) Similar imaging from a *Egr2^{Cre};Phox2b^{27AlaCKI}* neonate. Note the considerable loss of *Phox2b*-expressing RTN neurons, as demonstrated previously in Ramanantsoa et al., 2011 and in Ruffault et al., 2015. Scale bars, 200 µm. Representative of 2 animals.

Supplementary Figure 11

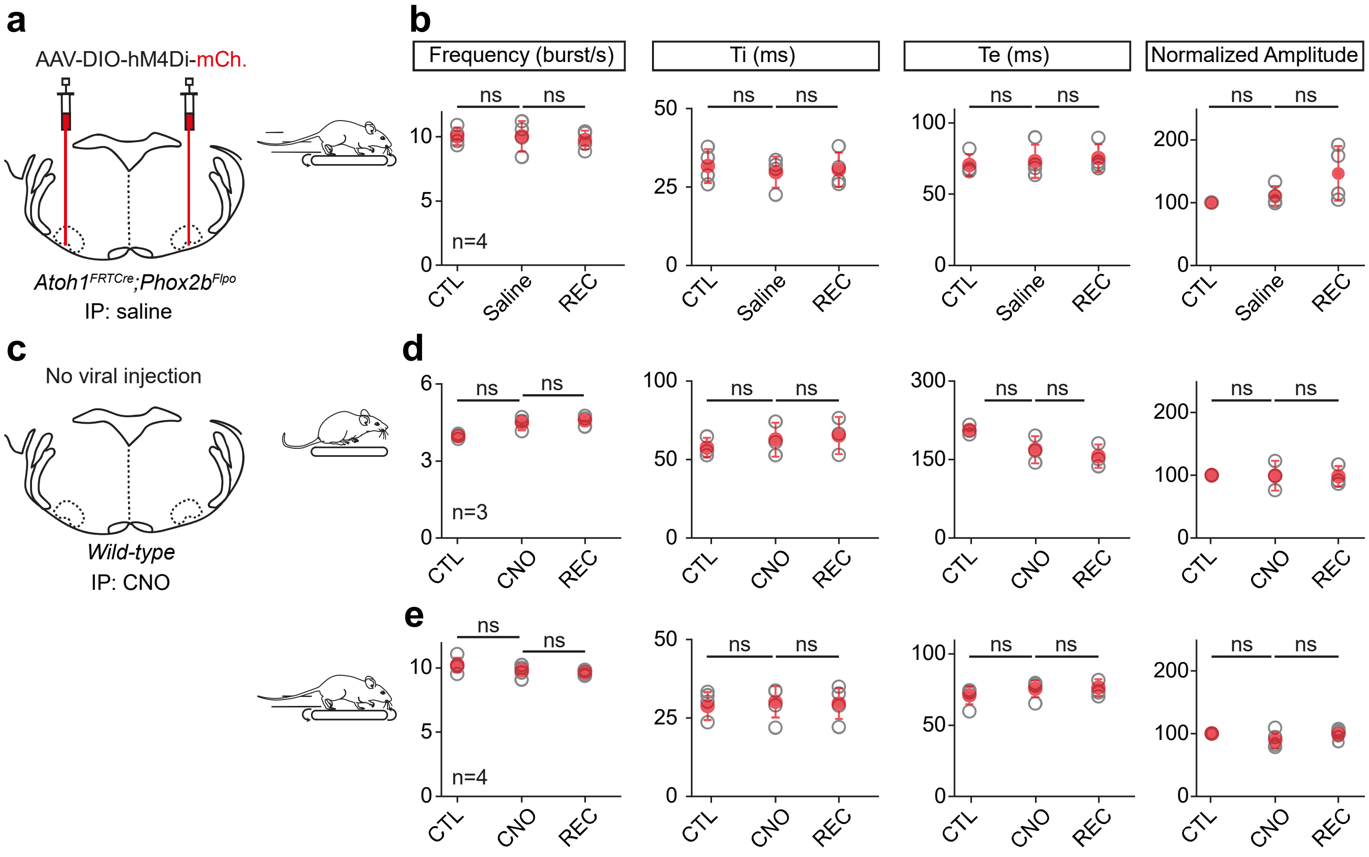

Supplementary Figure 11. Controls for chemogenetic silencing experiments. Related to Figure 6.
(a) Experimental strategy for the bilateral transfection of RTN$^{Phox2b/Atoh1}$ neurons using the inhibitory DREADD receptor hM4Di in *Atoh1*^{FRTCre}*;Phox2b*^{Flpo} adult mice. Injected mice were challenged to run on the motorized treadmill at 40 cm/s before, during and after intraperitoneal (IP) administration of saline solution.
(b) Quantifications of the diaphragm mean frequency, inspiratory (Ti) and expiratory (Te) times, and normalized amplitude before (CTL), during (Saline) and after (REC) IP administration of saline. Grey open circles are the means of individual mice, and filled red circles are the means ± SD across n mice. Saline administration alone does not alter the animals' capacity to modify its respiratory parameters during treadmill running. ns, not significant, paired t-tests. Exact p values are, from left to right: frequency: p > 0,9999; p = 0,3955; Ti: p = 0,3259; p = 0,7596; Te: p = 0,375; p = 0,625; amplitude: p = 0,3227; p = 0,8442.
(c) Experimental strategy to control for the effect of CNO administration alone, i.e., without expression of the hM4Di receptor, on breathing in resting and running mice.
(d, e) Similar respiratory parameters as in (**b**) during rest (**d**) and during treadmill running at 40 cm/s (**e**) before, during and after IP administration of CNO at 10 mg/kg. ns, not significant, paired t-tests. Exact p values are, from left to right in **d**: frequency: p = 0,1383; p < 0,0001; Ti: p = 0,2131; p = 0,221; Te: p = 0,1727; p = 0,0503; amplitude: p = 0,6895; p = 0,9188. From left to right in **e**: frequency: p = 0,0641; p = 0,4211; Ti: p = 0,4675; p = 0,5051; Te: p = 0,0625; p > 0,9999; amplitude: p = 0,5181; p = 0,2127.
For all graphs, source data are provided as a Source Data file.

Supplementary Figure 12

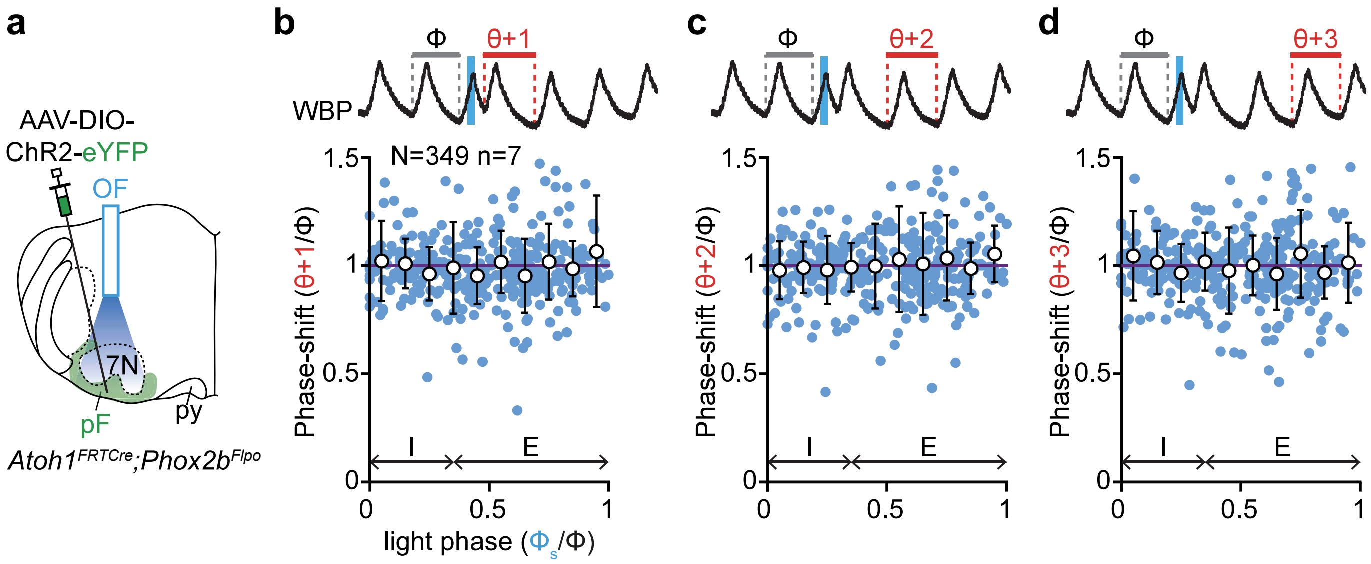

Supplementary Figure 12. Impact of photoactivating RTN$^{Phox2b/Atoh1}$ neurons on respiratory cycles following the light-perturbed cycle. Related to Figure 7.

(a) Experimental strategy for photoactivating RTN$^{Phox2b/Atoh1}$ neurons unilaterally in *Atoh1*^{FRTCre};*Phox2b*^{Flpo} adult mice.

(b) *Top*: whole body plethysmography (WBP) recording of respiratory cycles around a 50 ms stimulation. The cycle n+1 following the perturbed cycle is annotated on the trace (θ+1). *Bottom*: quantification of the perturbed cycle n+1 normalized to the control cycle (θ+1/Φ) as a function of the light phase (light cycle normalized to the control cycle: Φ_s/Φ). Values < 1 (purple line) indicate a shortening of the perturbed cycle (see Figure 2 for details). Blue circles represent individual data from N random trials from n mice and white circles are averages ± SD across all trials within 0.1 ms bins. Inspiration (I) and expiration (E) mean durations are annotated.

(c, d) Similar representations for cycles n+2 (θ+2, **c**) and n+3 (θ+3, **d**).

Source data are provided as a Source Data file.
