# Supplementary material for: Upregulation of breathing rate during running exercise by central locomotor circuits in mice
Source: Nat Commun. 2023 May 22;14:2939. doi: 10.1038/s41467-023-38583-6 (PMC10203288; doi:10.1038/s41467-023-38583-6)
Supplement: Supplementary file 2 — Reporting Summary [file 41467_2023_38583_MOESM2_ESM.pdf]

nature portfolio

Corresponding author(s): Julien Bouvier

Last updated by author(s): Mar 22, 2023

Reporting Summary

Nature Portfolio wishes to improve the reproducibility of the work that we publish. This form provides structure for consistency and transparency in reporting. For further information on Nature Portfolio policies, see our Editorial Policies and the Editorial Policy Checklist.

Statistics

For all statistical analyses, confirm that the following items are present in the figure legend, table legend, main text, or Methods section.

n/a	Confirmed	
☐	☒	The exact sample size (*n*) for each experimental group/condition, given as a discrete number and unit of measurement
☐	☒	A statement on whether measurements were taken from distinct samples or whether the same sample was measured repeatedly
☐	☒	The statistical test(s) used AND whether they are one- or two-sided<br>*Only common tests should be described solely by name; describe more complex techniques in the Methods section.*
☒	☐	A description of all covariates tested
☐	☒	A description of any assumptions or corrections, such as tests of normality and adjustment for multiple comparisons
☐	☒	A full description of the statistical parameters including central tendency (e.g. means) or other basic estimates (e.g. regression coefficient) AND variation (e.g. standard deviation) or associated estimates of uncertainty (e.g. confidence intervals)
☐	☒	For null hypothesis testing, the test statistic (e.g. *F*, *t*, *r*) with confidence intervals, effect sizes, degrees of freedom and *P* value noted<br>*Give P values as exact values whenever suitable.*
☒	☐	For Bayesian analysis, information on the choice of priors and Markov chain Monte Carlo settings
☒	☐	For hierarchical and complex designs, identification of the appropriate level for tests and full reporting of outcomes
☒	☐	Estimates of effect sizes (e.g. Cohen's *d*, Pearson's *r*), indicating how they were calculated

Our web collection on statistics for biologists contains articles on many of the points above.

Software and code

Policy information about availability of computer code

Data collection	2ndLOOK v2.0, ZEN v3.4, LAS X v3.5, LabSribe NI v3.0, NI MAX v19.6.
Data analysis	MATLAB R2021a, LabSribe v4, Excel, GraphPad Prism 7.

For manuscripts utilizing custom algorithms or software that are central to the research but not yet described in published literature, software must be made available to editors and reviewers. We strongly encourage code deposition in a community repository (e.g. GitHub). See the Nature Portfolio guidelines for submitting code & software for further information.

Data

Policy information about availability of data

All manuscripts must include a data availability statement. This statement should provide the following information, where applicable:

- Accession codes, unique identifiers, or web links for publicly available datasets
- A description of any restrictions on data availability
- For clinical datasets or third party data, please ensure that the statement adheres to our policy

The data that support the findings can be found in the Source Data provided with the paper. Original microscopy data have been deposited to Mendeley Data (http://dx.doi.org/10.17632/wpbyxgfr96.1). Source data are provided with this paper. This study did not generate any custom code.

Human research participants

Policy information about studies involving human research participants and Sex and Gender in Research.

Reporting on sex and gender	N/A
Population characteristics	N/A
Recruitment	N/A
Ethics oversight	N/A

Note that full information on the approval of the study protocol must also be provided in the manuscript.

Field-specific reporting

Please select the one below that is the best fit for your research. If you are not sure, read the appropriate sections before making your selection.

☒ Life sciences ☐ Behavioural & social sciences ☐ Ecological, evolutionary & environmental sciences

For a reference copy of the document with all sections, see nature.com/documents/nr-reporting-summary-flat.pdf

Life sciences study design

All studies must disclose on these points even when the disclosure is negative.

Sample size	Sample size is based both on the 3R principles and to allow statistical tests to be performed and to be informative and valid. sample size is indicated for all conditions.
Data exclusions	No data were excluded from the analyses.
Replication	All experiments were replicated n times (n being the sample size for each condition). Sample size vary accross experiments, but was at least of n=3. Sample size for each experiment is indicated in the figures and/or their legends.
Randomization	There is no randomization. Animals are allocated to a group depending on their genotype or treatment.
Blinding	The investigators were not blinded to group allocation during data collections and analysis, due to the nature of the experiments and the clear differences between groups. Indeed in most cases, experimental animals were identifiable by the position of the implant they carry, or showed a clear phenotypic response (photo-activations, silencing...) which was very specific to the structure targeted. Also, in most cases, analyses aim at comparing a resting state to a running state, or a control state to a photo-activation state, a transition that is induced by the experimenter and is detectable on the recording.

Reporting for specific materials, systems and methods

We require information from authors about some types of materials, experimental systems and methods used in many studies. Here, indicate whether each material, system or method listed is relevant to your study. If you are not sure if a list item applies to your research, read the appropriate section before selecting a response.

Materials & experimental systems

n/a	Involved in the study	
☐	☒	Antibodies
☒	☐	Eukaryotic cell lines
☒	☐	Palaeontology and archaeology
☐	☒	Animals and other organisms
☒	☐	Clinical data
☒	☐	Dual use research of concern

Methods

n/a	Involved in the study	
☒	☐	ChIP-seq
☒	☐	Flow cytometry
☒	☐	MRI-based neuroimaging

Antibodies

Antibodies used	PRIMARY ANTIBODIES:<br>goat anti-ChAt # AB144P, Merck Millipore<br>chicken anti-GFP # 1020, Aves Labs

	rabbit anti-RFP # 600-401-379, Rockland<br>rabbit anti-SST # T-4103, BMA Biomedicals<br>sheep anti-TH # AB1542, Merck Millipore.<br>(all are listed in the method section)<br><br>SECONDARY ANTIBODIES:<br>All are affinity-purified antibodies obtained from Jackson ImmunoResearch, made in donkey, and used at a final dilution of 1:500:<br>donkey anti-chicken AlexaFluor 488 (ref. # 703-545-155)<br>donkey anti-rabbit Cy3 (ref. # 711-165-152) or Cy5 (ref. # 711-175-152)<br>donkey anti-Goat AlexaFluor 647 (ref. # 705-605-147)<br>donkey anti-sheep Cy 3 (ref. # 713-166-147).<br>(all are listed in the method section)
Validation	PRIMARY ANTIBODIES:<br>goat anti-ChAt # AB144P, Merck Millipore:<br>Anti-Choline Acetyltransferase Antibody detects level of ChAT and has been published and validated for use in IH(P), IC, IH and WB.<br>PumMed ID: 26053681, 26052670, 26042202<br><br>Chicken anti-GFP # 1020, Aves Labs. From the supplier: "Antibodies were analyzed by western blot analysis (1:5000 dilution) and immunohistochemistry (1:500 dilution) using transgenic mice expressing the GFP gene product. Immunohistochemistry used tetramethyl rhodamine-labeled anti-chicken IgY".<br>PubMed ID: 36543133, 36530170, 36525974, 36522498 and more than 600 others.<br><br>Rabbit anti-RFP # 600-401-379, Rockland. From the supplier: "The immunogen is a Red Fluorescent Protein (RFP) fusion protein corresponding to the full length amino acid sequence (234aa) derived from the mushroom polyp coral Discosoma".<br>PubMed ID: 35705049, 35191834, 35681062, 35715418 and more than 100 others.<br><br>Rabbit anti-SST # T-4103, BMA Biomedicals. From the supplier: "This antibody was generated by immunization of rabbits with Somatostatin-14 coupled to a carrier protein. This antibody has been tested and validated in immunohistochemistry (IHC)".<br><br>Sheep anti-TH # AB1542, Merck Millipore. From the supplier: "Anti-Tyrosine Hydroxylase Antibody is an antibody against Tyrosine Hydroxylase for use in IH & WB. Immunohistochemistry (peroxidase): A previous lot of this antibody was used at 1:1,000 dilution. The antibody gives specific labeling of noradrenergic axons in primate cerebral cortex. (Brain Res., 1989, 500:313-324.). Quality Assurance: Routinely evaluated by Western Blot on mouse brain lysates.".<br>PubMed ID: 25716845, 25740518, 25664911, 26419281 and many more.

Animals and other research organisms

Policy information about studies involving animals; ARRIVE guidelines recommended for reporting animal research, and Sex and Gender in Research

Laboratory animals	Mice were used. Both wild-type and genetically-modified mice, all on the C57BL/6J background. Genetically-modified lines were:<br>- Vglut2Cre,<br>- Ai32(RCL-ChR2(H134R)/EYFP)<br>- Egr2Cre<br>- Phox2b27AlaCKI<br>- Atoh1FRTCre; Phox2bFlpo<br><br>Experiments were performed on animals of either sex, aged 2 to 3 months at the time of first injection.<br><br>Animals were housed in the licensed animal facility of NeuroPSI. They were group-housed with free access to food and water in controlled temperature (21°C) and humidity (between 40 and 55%) conditions and exposed to a conventional 12-h light/dark cycle.
Wild animals	No wild animals were used in the study.
Reporting on sex	Considering that sex differences are not expected for the motor interactions investigated here, and that ethic regulations constrain the number of animals in experimental series, we used animals irrespective their gender and obtained consistent results across all animals. Data from males and females were pooled.
Field-collected samples	No field-collected samples were used in the study.
Ethics oversight	All procedures were approved by the French Ethical Committee ("Comité d'éthique en Expérimentation Animale", CEEA #59, authorization 2020-022410231878) and conducted in accordance with EU Directive 2010/63/EU.

Note that full information on the approval of the study protocol must also be provided in the manuscript.
